# Melphalan induces cardiotoxicity through oxidative stress in cardiomyocytes derived from human induced pluripotent stem cells

**DOI:** 10.1186/s13287-020-01984-1

**Published:** 2020-11-05

**Authors:** Rui Liu, Dong Li, Fangxu Sun, Antonio Rampoldi, Joshua T. Maxwell, Ronghu Wu, Peter Fischbach, Sharon M. Castellino, Yuhong Du, Haian Fu, Anant Mandawat, Chunhui Xu

**Affiliations:** 1 Department of Pediatrics, Emory University School of Medicine and Children’s Healthcare of Atlanta, 2015 Uppergate Drive, Atlanta, GA 30322 USA; 2 Department of Pediatrics, The Third Xiangya Hospital of Central South University, Changsha, 410013 Hunan China; 3 School of Chemistry and Biochemistry and the Petit Institute for Bioengineering and Bioscience, Georgia Institute of Technology, Atlanta, GA 30332 USA; 4 Emory Chemical Biology Discovery Center and the Department of Pharmacology and Chemical Biology, Emory University School of Medicine, Atlanta, GA 30322 USA; 5 Department of Medicine, Emory University School of Medicine, Atlanta, GA 30322 USA; 6 Department of Hematology and Medical Oncology, Emory University School of Medicine, Atlanta, GA 30322 USA; 7 Cardio-Oncology Program, Winship Cancer Institute of Emory University, Atlanta, GA 30322 USA; 8 Wallace H. Coulter Department of Biomedical Engineering, Georgia Institute of Technology and Emory University, Atlanta, GA 30322 USA

**Keywords:** Cardiotoxicity, Chemotherapy, Contractility, Oxidative stress, Stem cells

## Abstract

**Background:**

Treatment-induced cardiotoxicity is a leading noncancer-related cause of acute and late onset morbidity and mortality in cancer patients on antineoplastic drugs such as melphalan—increasing clinical case reports have documented that it could induce cardiotoxicity including severe arrhythmias and heart failure. As the mechanism by which melphalan impairs cardiac cells remains poorly understood, here, we aimed to use cardiomyocytes derived from human induced pluripotent stem cells (hiPSC-CMs) to investigate the cellular and molecular mechanisms of melphalan-induced cardiotoxicity.

**Methods:**

hiPSC-CMs were generated and treated with clinically relevant doses of melphalan. To characterize melphalan-induced cardiotoxicity, cell viability and apoptosis were quantified at various treatment durations. Ca^2+^ transient and contractility analyses were used to examine the alterations of hiPSC-CM function. Proteomic analysis, reactive oxygen species detection, and RNA-Sequencing were conducted to investigate underlying mechanisms.

**Results:**

Melphalan treatment of hiPSC-CMs induced oxidative stress, caused Ca^2+^ handling defects and dysfunctional contractility, altered global transcriptomic and proteomic profiles, and resulted in apoptosis and cell death. The antioxidant *N*-acetyl-l-cysteine attenuated these genomic, cellular, and functional alterations. In addition, several other signaling pathways including the p53 and transforming growth factor-β signaling pathways were also implicated in melphalan-induced cardiotoxicity according to the proteomic and transcriptomic analyses.

**Conclusions:**

Melphalan induces cardiotoxicity through the oxidative stress pathway. This study provides a unique resource of the global transcriptomic and proteomic datasets for melphalan-induced cardiotoxicity and can potentially open up new clinical mechanism-based targets to prevent and treat melphalan-induced cardiotoxicity.

## Background

Chemotherapeutic drug-induced cardiotoxicity has emerged as a leading noncancer-related cause of morbidity and mortality in long-term cancer survivors in both adults and children [1, 2]. In particular, melphalan, a cytotoxic alkylating agent used in treatment for malignancies such as multiple myeloma, leukemia, and ovarian cancer [3–5], could induce cardiac complications including supraventricular tachycardia, atrial fibrillation, ventricular tachycardia, and left ventricular heart failure [6, 7]. A retrospective analysis found that 11% of the patients receiving melphalan prior to bone marrow transplantation developed a supraventricular tachycardia, with 73% being atrial fibrillation or atrial flutter [8]. Another study indicated that a rapid ventricular rate was associated with 91.6% of the patients who developed atrial fibrillation related to melphalan treatment [9]. However, it remains unknown how melphalan causes the adverse cardiac effects. Hence, since melphalan is a mainstay treatment for several malignancies and for bone marrow transplantation conditioning regimens, it is necessary to study the mechanism of melphalan-induced cardiotoxicity so that targeted treatment can be developed to ameliorate its cardiotoxicity.

Traditionally, studies on drug-induced toxicity have mainly relied on animal models [10]. However, these models do not always predict human response to drugs [11], mainly due to physiological differences from human cardiomyocytes (CMs), which lead to different mechanisms of actions. The use of human primary CMs would be the ideal choice for cardiotoxicity testing; however, these cells are difficult to obtain and possess limited growth capacity. There is a need to develop a new physiologically relevant model that can reliably be used to reproduce drug-induced cardiotoxicity. Human induced pluripotent stem cell-derived cardiomyocytes (hiPSC-CMs) could be a valuable asset to enhance data previously obtained from studies with animal models and primary CMs [12, 13]. Due to their self-renewal capacity and differentiation potential in vitro, hiPSCs can provide an unlimited supply of physiologically relevant CMs [14]. Indeed, hiPSC-CMs have been successfully used to evaluate drug-induced cardiotoxicity from anthracyclines, trastuzumab, and tyrosine kinase inhibitors [15–18].

To determine the potential cardiac toxicities induced by melphalan, the present study was conducted to characterize the effects of melphalan on hiPSC-CMs. Specifically, this cardiotoxicity study focused on (1) characterization of the melphalan-caused alterations at molecular, cellular, and functional levels based on cell survival, Ca^2+^ handling, contractility, and expression of the genes related to these processes; (2) identification of underlying mechanisms using proteomic and RNA-Sequencing (RNA-Seq) analyses; and (3) exploration of promising treatment strategies to ameliorate the side effects induced by melphalan.

## Methods

### Sources of reagents

Vendor information and catalog numbers for major reagents are available in Table S1.

### Cardiomyocyte differentiation

Two hiPSC lines SCVI-273 (Stanford Cardiovascular Institute) and IMR90 (WiCell Research Institute) were fed daily with mTeSR1-defined medium. For CM differentiation, hiPSCs were induced using a small molecule-guided differentiation protocol with CHIR99021 and IWR1 [19]. hiPSC-CMs were further enriched by the metabolic selection method from differentiation day 11 to 14 [20]. Alternatively, enriched hiPSC-CMs were generated by microscale generation of cardiospheres at differentiation day 6 [21]. Cells used in proteomic analysis were prepared by the enrichment of hiPSC-CMs through cardiosphere generation; cells used in other experiments were prepared by metabolic selection. Cells were observed under a microscope daily for beating cells, which typically appeared by day 7–9.

### Immunocytochemistry and cardiomyocyte purity assay

hiPSC-CMs were fixed in 4% PFA for 15 min and permeabilized in ice-cold methanol for 2 min at room temperature (RT). The cells were then blocked with 5% NGS in PBS at RT for 1 h and incubated with primary antibodies (Table S2) in 3% NGS overnight at 4 °C in dark. Then, the cells were incubated with the corresponding secondary antibodies at RT for 1 h in dark followed by counterstaining the nuclei with 7 μM Hoechst. Imaging was performed using an inverted microscope (Axio Vert.A1). Differentiation cultures were analyzed for CM purity using antibodies against NKX2-5, a cardiac-specific transcription factor. Images were acquired and quantitatively analyzed using ArrayScan XTI Live High Content Platform (Thermo Fisher Scientific) with mask modifiers for NKX2-5 restricted to the nucleus [22].

### Preparation of melphalan

The stock solution of 10 mM melphalan was prepared by dissolving the drug in DMSO and stored at − 80 °C. Treatment refreshing frequency of 24 h was selected due to the half-life of melphalan being approximately 75 min [23]. On the day of experiment, the drug stock solution was further diluted in the culture medium to 2× test concentrations, which was added to wells with hiPSC-CMs already containing the same volume of culture medium, finally reaching the intended test concentrations containing no more than 0.2% DMSO.

### Preparation of *N*-acetyl-l-cysteine (NAC)

The stock solution of 200 mM NAC was prepared by dissolving the drug in distilled water and stored at − 80 °C. Supplementation refreshing frequency of every single day was selected due to the half-life of NAC is around 5.6 h [24]. On the day of experiment, the drug stock solution was further diluted in the culture medium to 2× test concentration, which was added to wells with hiPSC-CMs ahead of adding the same volume of culture medium containing 2× test concentrations of melphalan, finally reaching the intended test concentrations of both drugs.

### Detection of cell viability and ATP content

Cell viability was measured using the CellTiter-Blue Cell Viability Assay, and ATP content was measured using the CellTiter-Glo 3D Cell Viability Assay per the manufacturer’s instructions.

### Detection of cell apoptosis

Cells were incubated with 5 μM CellEvent Caspase-3/7 Green Detection reagent and 7 μM Hoechst working solution in warm PBS with 5% fetal bovine serum for 30 min at 37 °C. Images were acquired and quantitatively analyzed using ArrayScan XTI Live High Content Platform with mask modifiers for caspase-3/7 restricted to the nucleus.

### Ca^2+^ transient assay

hiPSC-CMs at low densities were stained with 5 μM Fluo-4 AM in 1× normal Tyrode solution [22]. Dynamic fluorescence images were recorded using the ImageXpress Micro XLS System (Molecular Devices) at a frequency of 5 Hz for 12 s with × 20 magnification. The fluorescence intensities over time for individual cells were analyzed through MetaXpress software (Molecular Devices) by measurements in the region of interest. Ca^2+^ transient parameters were quantified using Clampfit software (pCLAMP 10.6).

### RNA extraction and quantitative real-time polymerase chain reaction (qRT-PCR)

RNA was extracted from about 10^6^ cells using Aurum total RNA mini kit. For qRT-PCR, 1 μg of RNA was reverse transcribed into cDNA using SuperScript VILO cDNA Synthesis Kit per the manufacturer’s instructions. qRT-PCR was performed on Applied Biosystems 7500 real-time PCR systems using the iTaq SyBr green master mix. Human-specific PCR primers (Table S3) for the genes examined were retrieved from open access websites (https://pga.mgh.harvard.edu/primerbank/). All samples were normalized to the level of the housekeeping gene *GAPDH*. Relative expression levels were calculated by the 2-∆∆Ct method.

### Proteomic analysis

Proteins were extracted from 3 to 4 × 10^6^ hiPSC-CMs per sample by resuspending the cells in the lysis buffer (50 mM HEPES pH = 7.4, 150 mM NaCl, 0.5% SDC, 10 units/mL benzonase, and 1 tablet/10 mL protease inhibitor) at 4 °C for 45 min. The protein concentration was determined by the BCA assay, and proteins in all samples were then normalized based on their concentrations. Proteins were digested and purified as described previously [25]. Tandem mass tag-labeling LC-MS/MS analyses, database search, data filtering, peptide quantification, and bioinformatic analysis were conducted as described previously [25]. Proteins were considered being up- or downregulated when the abundance changed by > 1.5-fold between two groups and the *P* values were < 0.05. Gene Ontology (GO) enrichment was performed with Database for Annotation, Visualization and Integrated Discovery [26]. GO terms with *P* values < 0.05 were considered significantly enriched by differentially expressed genes (DEGs).

### Detection of reactive oxygen species (ROS)

For intracellular and mitochondrial ROS detection, hiPSC-CMs were incubated with 12.5 μM carboxy-H_2_DCFDA and 7 μM Hoechst working solution in warm Live Cell Imaging Solution for 30 min at 37 °C, or 1 μM MitoSOX Red and 7 μM Hoechst working solution for 15 min. Images were acquired and analyzed using ArrayScan XTI Live High Content Platform with mask modifiers for MitoSOX and DCFDA restricted to the cytoplasm.

### Video-based analysis of contractility

Cells were plated at a density of 3 × 10^3^ cells/mm^2^ and cultured to form a sheet of spontaneous beating cells in each well. Beating was recorded using a phase-contrast inverted microscope (Axio Vert.A1) equipped with Zeiss AxioCam digital camera system, 30 s for each sample. Video-based analysis of contractility parameters was performed with Matlab (R2019a) algorithm by motion tracking function [27].

### RNA-Seq analysis

RNA-Seq analysis was performed at Novogene Corporation Inc. using Illumina TruSeq technology. RNA sequence reads were aligned to the human reference genome (GRCh38). HTSeq v0.6.1 was used to count the read numbers mapped of each gene, and then, Fragments Per Kilobase Million (FPKM) was calculated to estimate gene abundance. Differential expression analysis was performed using the DESeq2 R package (2_1.6.3). The resulting *P* values were adjusted using the Benjamini and Hochberg’s approach. Genes were considered being up- or downregulated when the abundance changed by > 2-fold between two groups and the adjusted *P* value was < 0.01. The Venn diagrams were prepared using the function vennDiagram in R based on the lists of genes with FPKM > 1. GO and KEGG pathway enrichment analyses of DEGs were implemented by the clusterProfiler R package. Corrected *P* values < 0.05 were considered significantly enriched by DEGs for both of GO terms and KEGG pathways.

### Statistics and data presentation

Data were analyzed in Excel or R and graphed in GraphPad Prism 7.04. Data are presented as mean ± SD. Comparisons were conducted via one-way ANOVA test followed by multiple comparison procedures (Dunnett’s method), two-sided chi-square test, or via an unpaired, two-tailed Student’s *t* test with significant differences defined by *P* < 0.05 (*), *P* < 0.01 (**), *P* < 0.001 (***), and *P* < 0.0001 (****). Sample sizes were given for each experiment. hiPSC line SCVI-273 derived CMs were used for qRT-PCR, proteomic, and RNA-Seq analyses. Both of hiPSC lines SCVI-273 and IMR90 derived CMs were used for the remaining experiments. Data from both hiPSC lines were combined for Ca^2+^ transient and contractility assays. Data from hiPSC line SCVI-273 are presented for other experiments.

## Results

### Melphalan treatment induces cell death and apoptosis in hiPSC-CMs

**Fig. 1:**
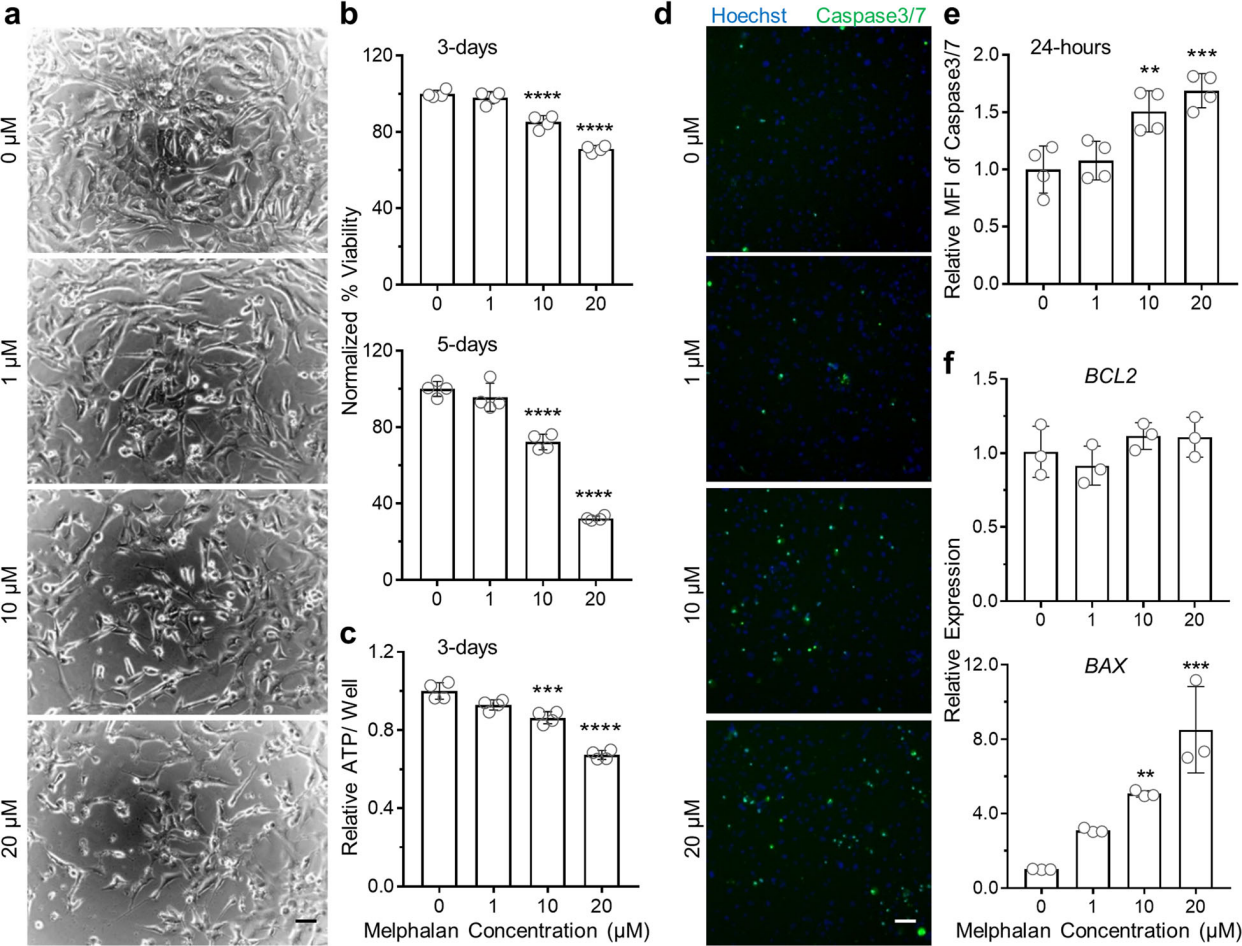
Melphalan treatment induces cell death and apoptosis in hiPSC-CMs. **a** Representative images of hiPSC-CMs treated with melphalan for 5 days. Scale bar, 40 μm. **b** Measurement of cell viability by CellTiter-Blue Viability assay in hiPSC-CMs treated with melphalan for 3 and 5 days, respectively (*n* = 4). **c** Quantification of ATP content/well which indirectly showed viability by CellTiter-Glo 3D Viability assay in hiPSC-CMs treated with melphalan for 3 days (*n* = 4). **d**, **e** Representative images and quantification of cell apoptosis in hiPSC-CMs upon melphalan treatment for 24 h by CellEvent Caspase-3/7 Green Detection reagent and Hoechst staining (*n* = 4). Cells positive for activated caspase-3/7 emitted bright green nuclear fluorescence. Scale bar, 50 μm. **f** qRT-PCR analysis showing relative gene expression levels of apoptosis-related genes *BCL2* and *BAX* in hiPSC-CMs treated with melphalan for 3 days (*n* = 3). The viability and relative MFI were normalized by the average values of no melphalan group. Comparisons were conducted between each treatment group and no melphalan group via one-way ANOVA test. **P value < 0.01; ***P value < 0.001; ****P value < 0.0001

To investigate the cardiotoxicity of melphalan, we generated enriched hiPSC-CMs (Fig. S1) and treated them with melphalan at 4 doses ranging from 0 to 20 μM; the highest dose was slightly above the *C*_max_ of melphalan (15.4 μM) [23]. hiPSC-CMs exposed to 20 μM melphalan contracted weakly after 24 h compared with other groups. After 48 h of treatment, many cells treated with 20 μM melphalan stopped contracting with many turning into round shape and detaching from the plate surface, indicating cell dysfunction and death. Cells treated with 10 μM melphalan presented similar morphology during 3 to 5 days. As shown in Fig. 1a, fewer cells remained following the treatment with 10 and 20 μM melphalan for 5 days.

In order to quantify the cell death, we first validated and optimized two cell viability assays, CellTiter-Blue and CellTiter-Glo 3D Cell Viability Assays, which were reliable and sensitive for the estimation of cell numbers of hiPSC-CMs (Fig. S2). Next, we examined cell viability in cultures after 3- and 5-day melphalan treatment. Based on CellTiter-Blue Cell Viability Assay, 10 μM melphalan treatment for 3 days caused a 15% loss of cells compared with no melphalan treatment and 20 μM melphalan treatment caused a 29% loss. When the treatment duration extended to 5 days, melphalan treatment exacerbated the cell loss, which increased to 28% for 10 μM and 68% for 20 μM (Fig. 1b). The dose-dependent cell death induced by melphalan was validated by CellTiter-Glo 3D Cell Viability Assay (Fig. 1c).

To evaluate if the reduced cell viability in melphalan-treated hiPSC-CMs was associated with apoptosis at the early stage, we treated hiPSC-CMs with various doses of melphalan for 24 h and measured activated caspases 3 and 7. As shown in Fig. 1d, e, relative mean fluorescence intensity (MFI) of caspase 3/7 significantly elevated in cells exposed to melphalan in a dose-dependent manner. To further confirm this phenomenon, we examined the expression of apoptosis-related genes by qRT-PCR in cells exposed to melphalan for 3 days. The level of anti-apoptosis gene *BCL2* detected was similar in all the groups, but the level of pro-apoptosis gene *BAX* detected was 5 times higher in hiPSC-CMs treated with 10 μM melphalan compared with no melphalan treatment and 8 times higher in hiPSC-CMs treated with 20 μM melphalan (Fig. 1f).

### Melphalan treatment of hiPSC-CMs results in Ca^2+^ handling defect and alters expression of genes encoding calcium channels and sarcomeric proteins

**Fig. 2:**
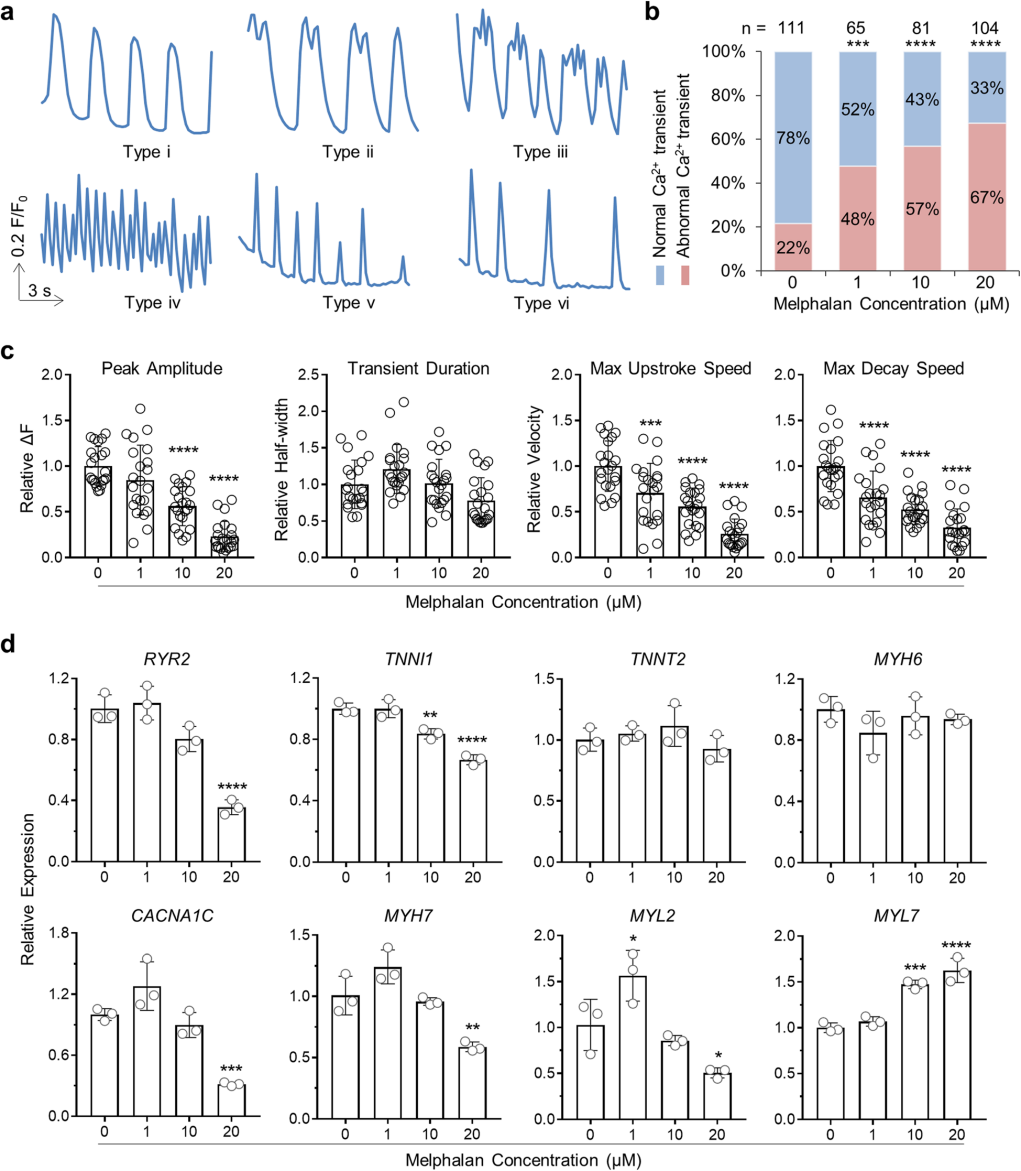
Melphalan treatment of hiPSC-CMs results in Ca^2+^ handling defect and alters expression of genes encoding calcium channels and sarcomeric proteins. **a** Representative traces showing intracellular Ca^2+^ transients in hiPSC-CMs treated with melphalan for 3 days. i, normal Ca^2+^ transients; ii–vi, abnormal Ca^2+^ transients. **b** Stacked bar charts showing percentage of CMs exhibiting normal (blue) or abnormal Ca^2+^ transients (red) under each condition. Sample sizes (*n*) were denoted at the top of each bar. **c** Quantification of peak amplitude, transient duration, maximum upstroke speed, and maximum decay speed of Ca^2+^ transients under each condition. Relative values were calculated based on the average values of the melphalan-treated group vs. untreated group (*n* = 22). **d** qRT-PCR panel showing relative gene expression levels of Ca^2+^ transporting-related genes including *RYR2* and *CACNA1C*, and CM structure-related genes including *TNNI1*, *TNNT2*, *MYH6/7*, and *MYL2/7* in hiPSC-CMs treated with melphalan for 3 days (*n* = 3). Relative expression values were calculated based on the average values of the melphalan-treated group vs. untreated group. Comparisons were conducted between each treatment group and no melphalan group via two-sided chi-square test for **b** or one-way ANOVA test for **c** and **d**. *P value < 0.05; **P value < 0.01; ***P value < 0.001; ****P value < 0.0001

Ca^2+^ is the critical link between electrical excitation and mechanical contraction. Carefully regulated transient rises and reductions of cytosolic Ca^2+^ correspond to the electrical signals that pervade the heart and control each cycle of contraction and relaxation of CMs. To investigate the effect of melphalan treatment on CM function, we assessed intracellular Ca^2+^ transients in hiPSC-CMs treated with various doses of melphalan for 3 days. In all conditions, as the representative traces shown in Fig. 2a, two categories of whole cell Ca^2+^ release events were observed: normal and abnormal Ca^2+^ transients. Cells were categorized as normal if the Ca^2+^ transients had mostly consistent amplitudes and rhythmicity, typical cardiac Ca^2+^ transient morphology (i.e., rapid upstroke and decay kinetics), and no obvious spontaneous Ca^2+^ release between transients (Fig. 2a (i)). Cells were categorized as abnormal if they exhibited oscillations of the diastolic Ca^2+^ signal (Fig. 2a (ii and iii)), unrecognizable single transient morphology (Fig. 2a (iv)), or notable inconsistent amplitudes or beat periods (Fig. 2a (v, vi)). Using these criteria, we counted the numbers of cells exhibiting normal or abnormal Ca^2+^ transients and calculated the proportion of each category for each culture condition (Fig. 2b). In hiPSC-CMs without melphalan treatment, the majority of the cells exhibited normal Ca^2+^ transients, whereas in hiPSC-CMs treated with melphalan, the percentage of cells exhibiting abnormal Ca^2+^ transients increased in a dose-dependent manner. Specifically, 48% of the cells showed abnormal Ca^2+^ transients when treated with 1 μM melphalan, 57% of the cells showed abnormal Ca^2+^ transients when treated with 10 μM melphalan, and 67% of the cells showed abnormal Ca^2+^ transients when treated with 20 μM melphalan. In addition, the treatment of hiPSC-CMs with melphalan at 10 and 20 μM significantly decreased Ca^2+^ transient amplitude without affecting Ca^2+^ transient duration compared with no melphalan treatment (Fig. 2c): the amplitude was reduced by 44% in cells exposed to 10 μM melphalan and 77% in cells exposed to 20 μM melphalan. The maximum upstroke and decay speeds of Ca^2+^ transients were also significantly decreased in melphalan-treated hiPSC-CMs (Fig. 2c): the maximum upstroke and decay speeds were reduced by 29–34% in cells exposed to 1 μM melphalan, 44–47% in cells exposed to 10 μM melphalan, and 67–74% in cells exposed to 20 μM melphalan. These observations suggest that exposure of hiPSC-CMs to melphalan results in intracellular Ca^2+^ handling dysfunction in a dose-dependent manner.

We next quantified the expression of genes encoding the components of calcium channels and sarcomere which are crucial to CM function by qRT-PCR in hiPSC-CMs under the above conditions (Fig. 2d). The expression of calcium channel proteins encoding genes *RYR2* and *CACNA1C* was reduced in cells treated with 20 μM melphalan compared with no melphalan treatment. The expression of *TNNI1* and *MYH7* was also lower in 10 and 20 μM melphalan-treated cells. The expression of light chain of myosin encoding genes *MYL2* decreased by 52% in 20 μM melphalan-treated cells but that of *MYL7* increased by 63%.

### Melphalan treatment alters protein expression levels of hiPSC-CMs identified by proteomic analysis

**Fig. 3:**
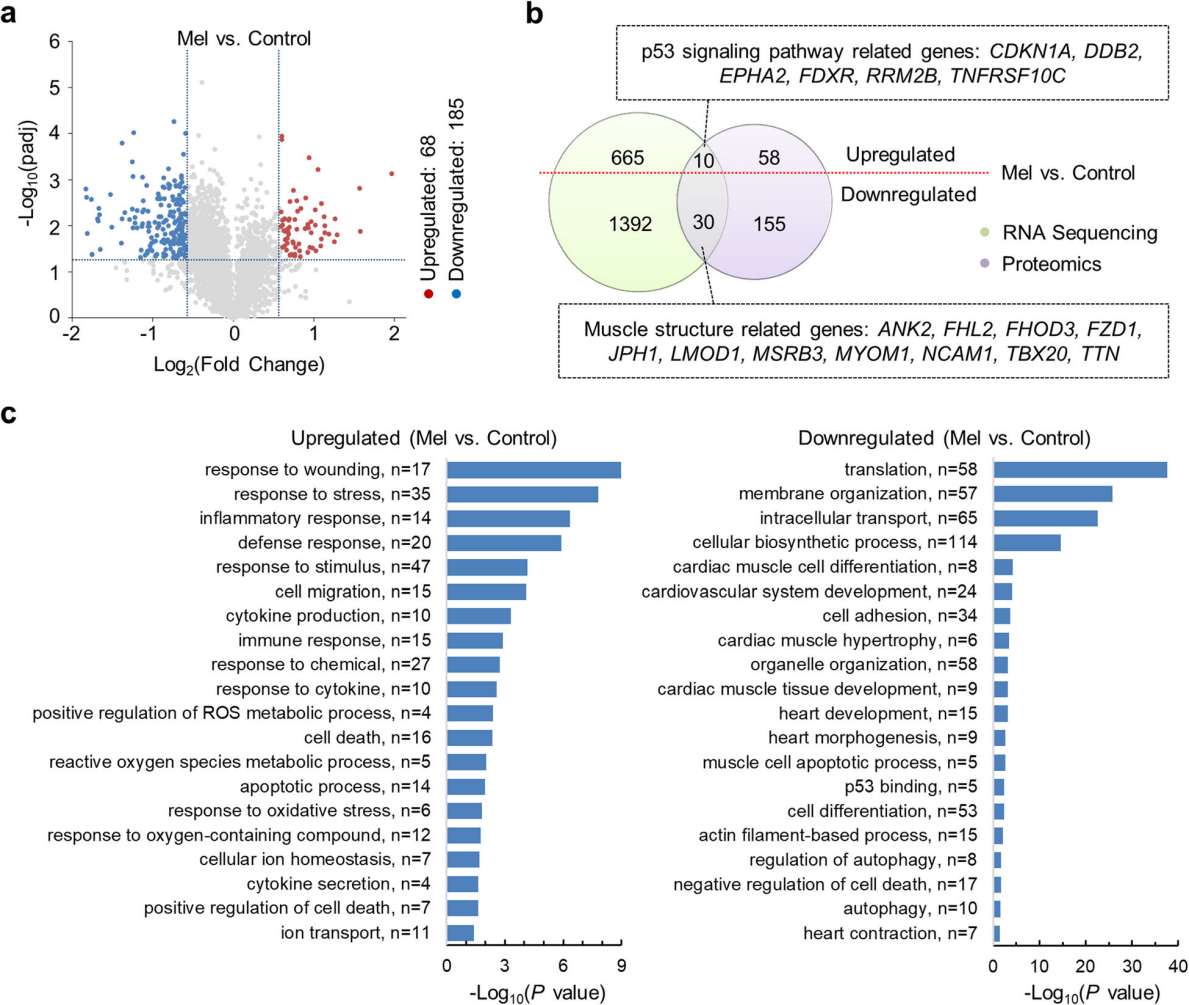
Melphalan treatment of hiPSC-CMs alters the expression of proteins identified by proteomic analysis. Proteomic analysis of hiPSC-CMs treated with 0 and 20 μM melphalan for 3 days (*n* = 3). **a** Volcano plot illustrating proteins with statistically significant differences in their abundance between control and melphalan-treated hiPSC-CMs. The log_2_(fold change) was plotted on the *x*-axis and the −log_10_(*P* value) on the *y*-axis (*P* value < 0.05 and fold change > 1.5). **b** Venn diagram showing the numbers of differentially expressed proteins identified by proteomics (purple circle) and genes identified by RNA-Seq (green circle). The red line divides areas into upregulated part and downregulated part. **c** Bar charts showing up- and downregulated proteins clustered by GO enrichment analysis. Length of bar indicates −log_10_(*P* value), and value of *n* denotes the count of involved proteins in each term. Control, no melphalan; Mel, 20 μM melphalan

To further evaluate the molecular alteration induced by melphalan and to investigate potential mechanisms of melphalan-induced cardiotoxicity, we treated hiPSC-CMs with or without 20 μM melphalan for 3 days and performed proteomic analysis to compare protein expression changes. Sixty-eight proteins were significantly upregulated and 185 downregulated in melphalan-treated hiPSC-CMs (Fig. 3a). GO analysis showed that melphalan treatment upregulated proteins associated with response to wounding, stress, and stimulus (Fig. 3c). The upregulation of proteins involved in apoptotic process and cell death was consistent with the aforementioned results based on cell viability and apoptosis detection at cellular level. More intriguingly, ROS seemed to play an important role due to several significantly enriched GO terms from the upregulated proteins, such as ROS metabolic process, response to oxidative stress, and response to oxygen-containing compound. In addition, the downregulated proteins were also related to cell adhesion, cardiovascular system development, actin filament-based process, and heart contraction (Fig. 3c).

### Melphalan treatment causes oxidative stress in hiPSC-CMs

**Fig. 4:**
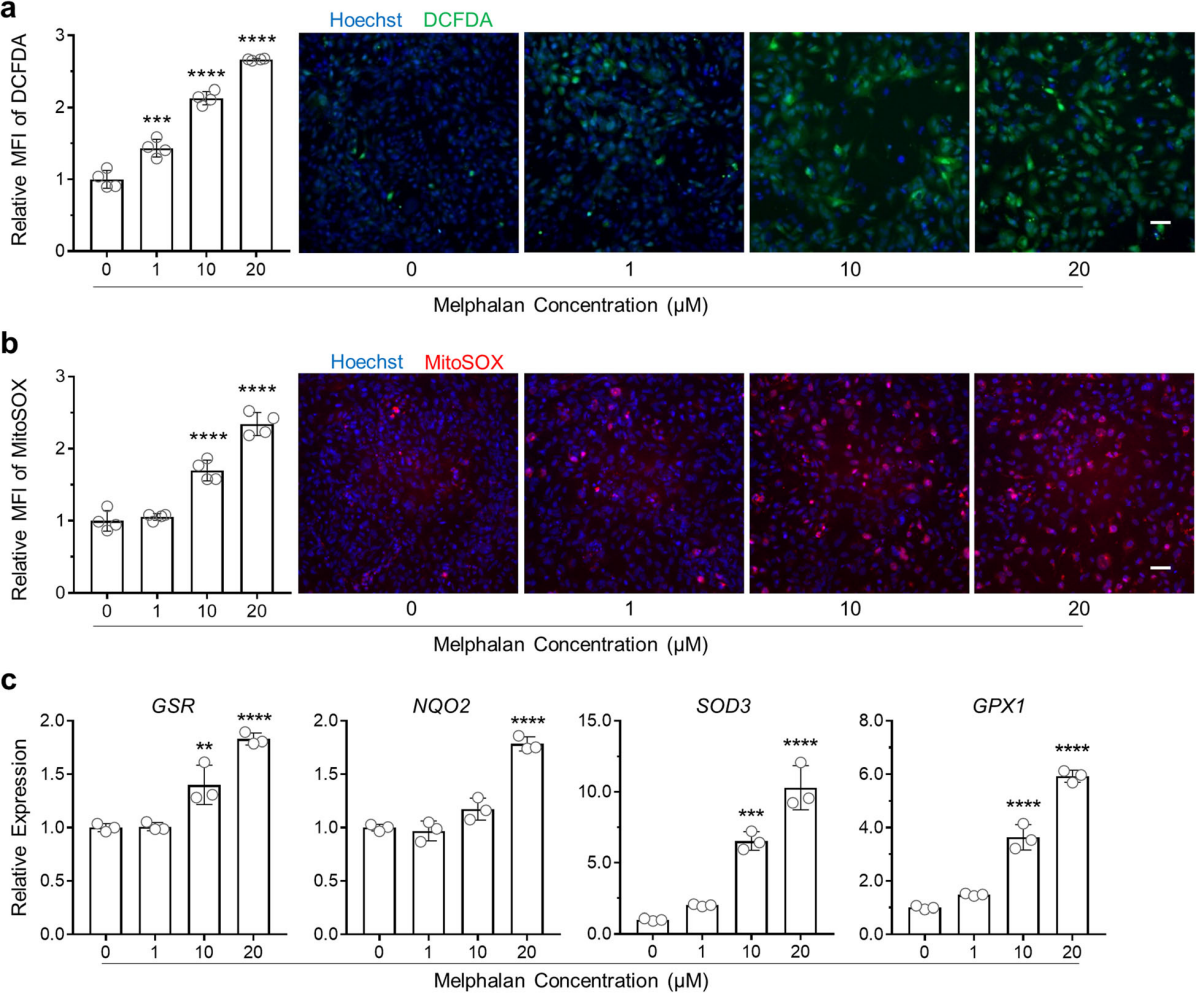
Melphalan treatment causes oxidative stress in hiPSC-CMs. **a** Representative images and quantification of intracellular ROS production in hiPSC-CMs treated with melphalan for 3 days via carboxy-H_2_DCFDA and Hoechst staining (*n* = 4). Scale bar, 50 μm. **b** Representative images and quantification of mitochondrial ROS production in hiPSC-CMs treated with melphalan for 3 days via MitoSOX and Hoechst staining (*n* = 4). Scale bar, 50 μm. **c** qRT-PCR analysis showing relative gene expression levels of oxidative stress-related genes including *SOD3*, *GSR*, *NQO2*, and *GPX1* in hiPSC-CMs treated with melphalan for 3 days (*n* = 3). Relative MFI and gene expression were calculated based on the average values of melphalan-treated group vs. untreated group. Comparisons were conducted between each treatment group and no melphalan group via one-way ANOVA test. **P value < 0.01; ***P value < 0.001; ****P value < 0.0001

To validate the finding from the proteomic experiments and the hypothesis that oxidative stress could be an underlying mechanism of cardiotoxicity caused by melphalan, we treated hiPSC-CMs with various doses of melphalan for 3 days and measured intracellular ROS by H_2_DCFDA probe and mitochondrial ROS by MitoSOX probe. As shown in Fig. 4a, increased ROS signals were detected in the cells treated with melphalan in a dose-dependent manner. The relative level of mitochondrial oxidative stress was 0.7 times higher in cells treated with 10 μM melphalan compared with no melphalan treatment, and 1.3 times higher in cells treated with 20 μM melphalan (Fig. 4b).

We next examined the expression of oxidative stress-related genes by qRT-PCR in hiPSC-CMs exposed to melphalan for 3 days. The expression of superoxide dismutase family of genes (*SOD1*, *SOD2*, and *SOD3*), reductase encoding genes (*PRDX5* and *NQO2*), and glutathione-related genes (*GSR*, *GPX1*) was significantly elevated in cells treated with 20 μM melphalan compared with no melphalan treatment (Fig. 4c, Fig. S3). Particularly, *SOD3* level detected was 5.5 times higher in cells exposed to 10 μM melphalan compared with no melphalan treatment and even higher (9.3 times) in cells treated with 20 μM melphalan. These results indicate that melphalan induces ROS production and increases oxidative stress in hiPSC-CMs in a dose-dependent fashion.

### NAC mitigates cell loss and mitochondrial ROS production in hiPSC-CMs under melphalan treatment

**Fig. 5:**
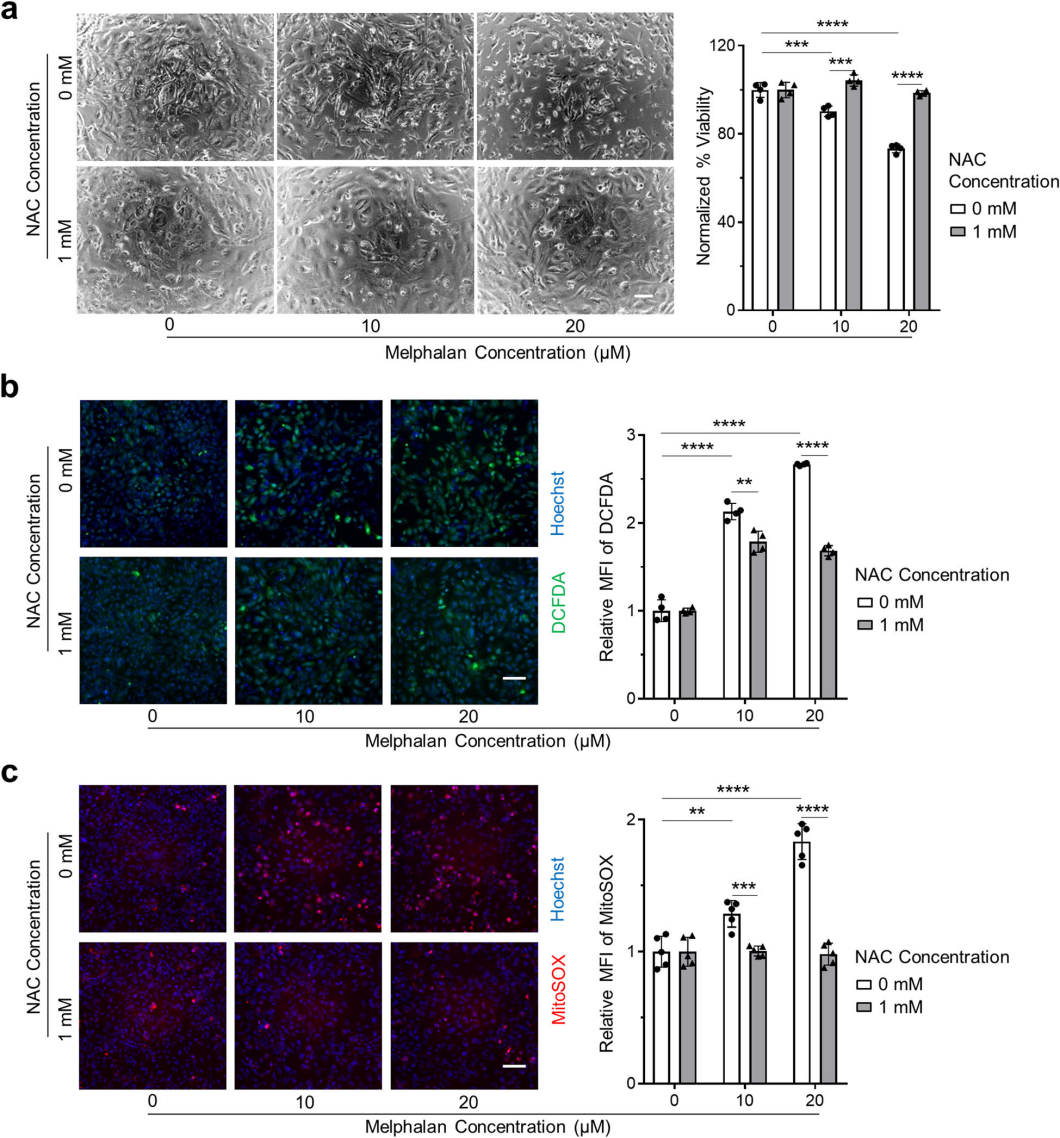
NAC mitigates the cell loss and mitochondrial ROS production in hiPSC-CMs under melphalan treatment. **a** Representative images and measurement of cell viability via CellTiter-Blue Viability Assay in hiPSC-CMs upon melphalan treatment with or without NAC supplementation for 3 days (*n* = 4). Scale bar, 40 μm. **b** Representative images and quantification of intracellular ROS production in hiPSC-CMs upon melphalan treatment with or without NAC supplementation for 3 days via carboxy-H_2_DCFDA and Hoechst staining (*n* = 4). Scale bar, 100 μm. **c** Representative images and quantification of mitochondrial ROS production in hiPSC-CMs upon melphalan treatment with or without NAC supplementation for 3 days via MitoSOX and Hoechst staining (*n* = 5). Scale bar, 100 μm. Normalization of viability and relative MFI was calculated based on the average values of melphalan-treated group vs. no melphalan groups. Comparisons were performed between the groups indicated via one-way ANOVA test or two-tailed Student’s *t* test. **P value < 0.01; ***P value < 0.001; ****P value < 0.0001

To further evaluate if ROS production plays a crucial role in melphalan-induced cardiotoxicity, we treated hiPSC-CMs with 0, 10, and 20 μM melphalan in combination with or without 1 mM of ROS scavenger NAC concomitantly, for 3 days, and measured cell viability and ROS production. The dose selection of NAC was based on previous studies in which 1 mM of NAC effectively attenuated the ethanol- and doxorubicin-induced oxidative stress in hiPSC-CMs [22, 28]. As shown in Fig. 5a, treatment of cells with NAC prevented the cell loss caused by melphalan treatment. Furthermore, NAC supplementation dramatically decreased intracellular ROS by 16% in 10 μM melphalan-treated hiPSC-CMs and 37% in 20 μM melphalan-treated hiPSC-CMs (Fig. 5b). More strikingly, treatment of cells with NAC mitigated mitochondrial oxidative stress caused by melphalan treatment to the level similar to that of no melphalan treatment (Fig. 5c). In addition, we observed that hiPSC-CMs exposed to melphalan with NAC supplementation contracted more powerfully and kept better morphology than those without NAC supplementation.

### NAC attenuates the alteration of hiPSC-CM beating indexes caused by melphalan treatment

**Fig. 6:**
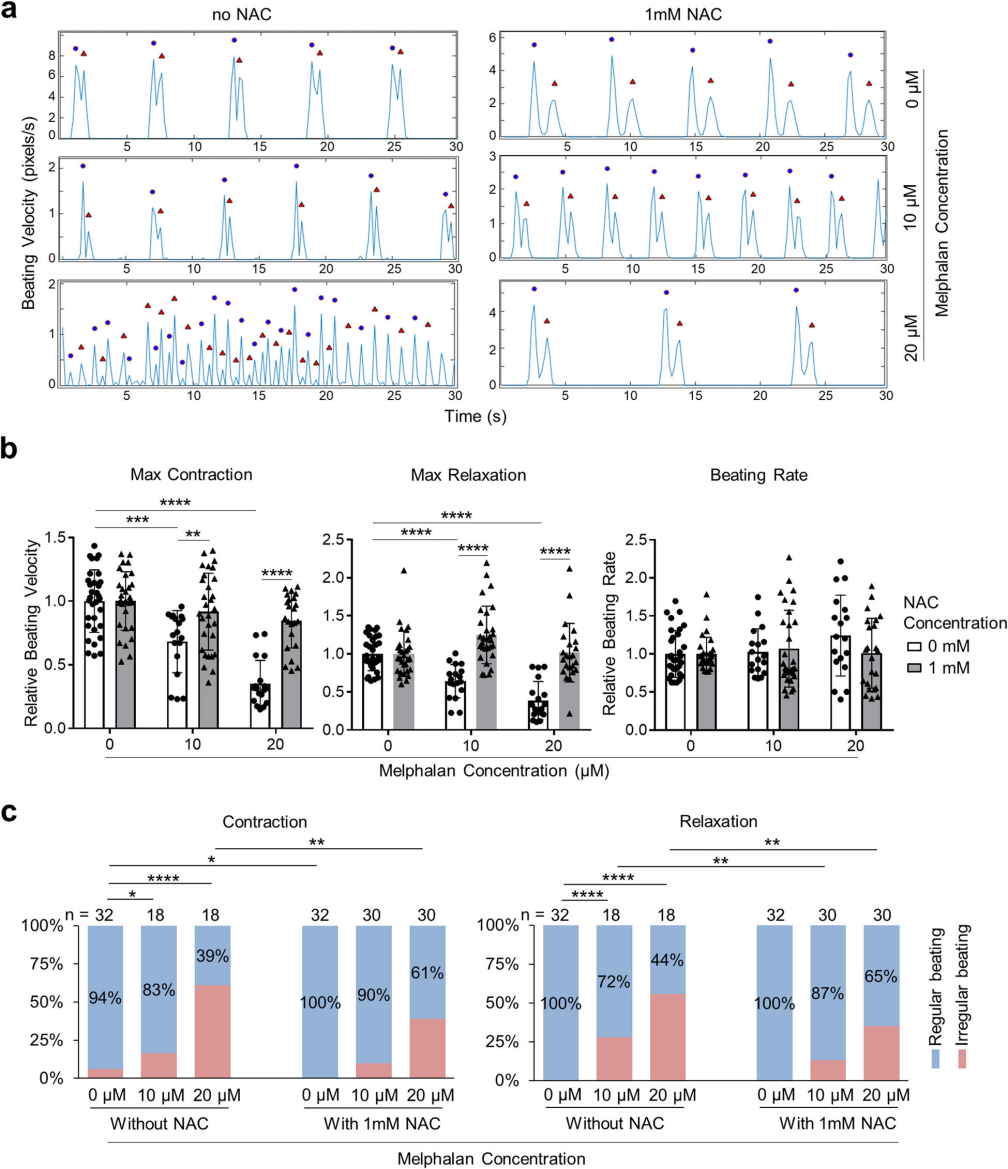
NAC ameliorates the alteration of cardiomyocyte beating indexes caused by melphalan. Analysis of hiPSC-CM contractility upon melphalan treatment with or without NAC supplementation for 3 days. **a** Representative traces showing the beating velocity recording of hiPSC-CMs. Blue dots denote contraction, and red triangles denote relaxation. **b** Quantification of maximum contraction, maximum relaxation, and beating interval changes. Relative values were calculated by dividing by the average beating velocity of no melphalan treatment groups, respectively (sample sizes were the same as **c**). **c** Stacked bar charts showing the percentage of wells of cells with regular (blue) or irregular (red) contractility pattern. Sample sizes (*n*) were given at the top of each bar. Comparisons were done between the groups indicated via one-way ANOVA test and two-tailed Student’s *t* test for **b**, or two-sided chi-square test for **c**. *P value < 0.05; **P value < 0.01; ***P value < 0.001; ****P value < 0.0001

Normal contraction and relaxation of CMs are essential to maintain normal organ function. To identify the influence of melphalan treatment and NAC supplementation on CM contractility, we recorded spontaneous beating and quantified beating indexes in hiPSC-CMs treated with 0, 10, and 20 μM melphalan with or without 1 mM NAC supplementation for 3 days. As shown in Fig. 6a, recorded traces presented the velocities of contraction and relaxation of each CM beating during 30-s periods under all conditions. We found that treatment of hiPSC-CMs with melphalan at 10 and 20 μM significantly decreased maximum contraction and maximum relaxation without distinct beating rate alteration compared with no melphalan treatment (Fig. 6b). Specifically, the maximum contraction and relaxation in cells exposed to 10 μM melphalan were reduced by 30–35%, which further dropped by 30% more in cells exposed to 20 μM melphalan. However, with 1 mM NAC supplementation, the maximum contraction and maximum relaxation in melphalan-treated cells retained nearly similar levels to the no melphalan treatment. These findings were consistent with microscopic observations of cell behaviors. In addition, we observed an increase in the incidence of irregular beating based on variation of contraction and relaxation velocity, from less than 6% in cells without melphalan treatment to 17–28% in cells treated with 10 μM melphalan and 56–61% in cells treated with 20 μM melphalan (Fig. 6c). NAC supplementation attenuated the degree of irregular beating caused by melphalan treatment: to 10–13% in the 10 μM melphalan-treated cells and 35–39% in the 20 μM melphalan-treated cells (Fig. 6c). Taken together, these results indicate that melphalan treatment of hiPSC-CMs impairs CM contractility, which could be ameliorated by NAC supplementation.

### NAC ameliorates melphalan-induced alteration of hiPSC-CM transcriptomic profiles characterized by RNA-Seq analysis

**Fig. 7:**
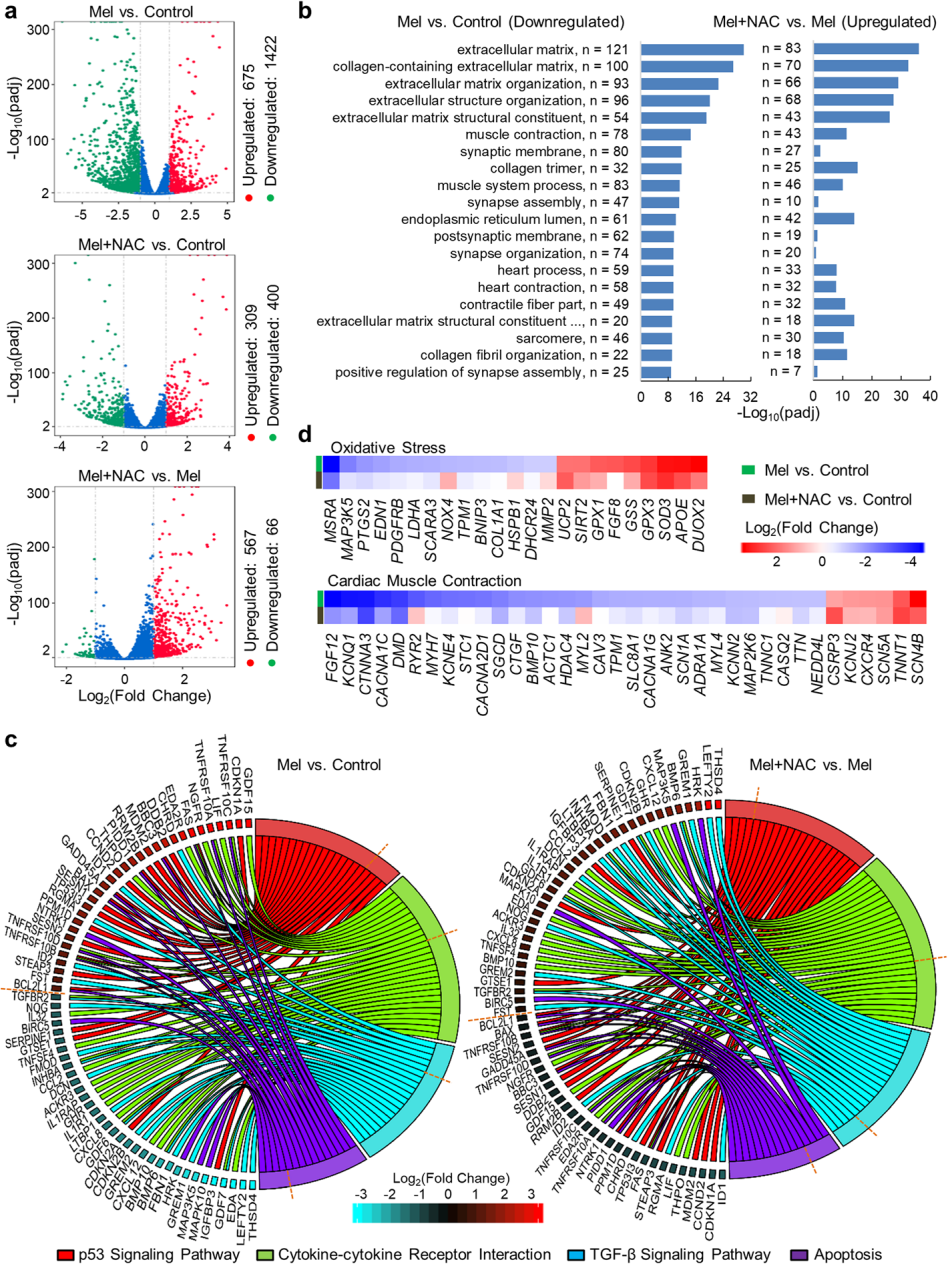
NAC attenuates melphalan-induced alteration of hiPSC-CM transcriptome profiles characterized by RNA-Seq analysis. RNA-Seq analysis of hiPSC-CMs upon 0 and 20 μM melphalan treatment with or without NAC supplementation for 3 days (*n* = 3). **a** Volcano plots presenting the DEGs when comparing any two groups. The up- or downregulated genes were identified based on padj < 0.01 and fold change > 2. **b** Bar charts showing top 20 downregulated GO terms in melphalan-treated hiPSC-CMs compared with control group, and the enrichment results of these GO terms in Mel+NAC-treated hiPSC-CMs compared with melphalan group. Length of bar indicates −log_10_(padj), and the value of *n* denotes the count of involved genes in each term. **c** Chord diagrams showing the DEGs of interested KEGG clusters in melphalan-treated hiPSC-CMs compared with control group, and the relative expression of these genes in Mel+NAC-treated hiPSC-CMs compared with melphalan group. In each chord diagram, KEGG pathways were presented on the right, and genes contributing to these enrichments were drawn on the left. Blue and red colors of displayed squares on the left indicate the levels of gene expression according to log_2_(fold change). The dark orange dashed lines were the boundary between up- and downregulated genes. **d** Heatmap showing the DEGs involved in GO terms of oxidative stress and cardiac muscle contraction in melphalan- or Mel+NAC-treated hiPSC-CMs compared with control group. Blue and red colors of displayed rectangles indicate the levels of gene expression according to log_2_(fold change). padj, adjusted *P* value; Control, no melphalan; Mel, 20 μM melphalan; Mel+NAC, 20 μM melphalan with 1 mM NAC

To further evaluate the molecular changes associated with melphalan-induced cardiotoxicity and rescue by NAC supplementation, we performed RNA-Seq to analyze global transcriptome profiles of hiPSC-CMs treated with vehicle (control group), 20 μM melphalan (Mel group), and 20 μM melphalan with 1 mM NAC (Mel+NAC group), respectively, for 3 days. As detected by RNA-Seq, 12,201 genes were commonly expressed in all three groups, and 309 genes were expressed in the control and Mel+NAC groups but not in the Mel group (Fig. S4a). As shown in Fig. 7a, treatment of the cells with melphalan resulted in up- and downregulation of 2097 genes (Mel vs. control), whereas NAC supplementation to melphalan-treated cells reduced the number of up- and downregulated genes to 709 (Mel+NAC vs. control). Interestingly, more genes were downregulated than upregulated by the treatment of melphalan (1422 vs. 675 in Mel vs. control), whereas NAC supplementation resulted in more genes being upregulated than downregulated (567 vs. 66 in Mel+NAC vs. Mel). As shown in Table S5, among the top 10 upregulated genes by melphalan treatment, 4 were direct p53 effectors (*CDKN1A*, *EDXR*, *TNFRSF10C*, and *GDF15*). Among the top 10 downregulated genes by melphalan treatment, 5 were correlated to cell adhesion (*CDH13*, *CNTN1*, *SDK1*, *CTNND2*, and *PARD3B*).

Given that more genes were downregulated by melphalan treatment and more genes were upregulated by NAC supplementation, we performed GO analysis of DEGs in these groups and examined the degree of the GO terms in these groups overlapped. As shown in Fig. 7b and Tables S5 and S6, melphalan treatment dramatically downregulated the expression of genes associated with extracellular matrix (121 genes), muscle contraction (78 genes), and synaptic membrane (80 genes). Interestingly, NAC supplementation upregulated many of the genes involved in these GO terms (extracellular matrix, 83 genes; muscle contraction, 43 genes; and synaptic membrane, 27 genes).

We also examined the signaling pathways regulated by melphalan treatment and NAC supplementation on the basis of KEGG enrichments (Table S5, S6). Noteworthily, several pathways were both regulated by melphalan treatment and NAC supplementation, including apoptosis pathway, p53 signaling, transforming growth factor (TGF)-β signaling, and cytokine-cytokine receptor interaction. As shown in Fig. 7c, the genes of apoptosis (e.g., *BAX* and *TNFRSF10C*) and p53 signaling pathway (e.g., *FAS* and *CDKN1A*) were mostly upregulated by melphalan (Mel vs. control), but they were mostly downregulated by NAC supplementation (Mel+NAC vs. Mel). Those in the TGF-β signaling pathway (e.g., *LEFTY2* and *THSD4*) and cytokine-cytokine receptor interaction (e.g., *BMP6* and *BMP10*) were mostly downregulated by melphalan treatment (Mel vs. control), but they were mostly upregulated by NAC supplementation (Mel+NAC vs. Mel).

In addition, we compared the regulation of genes involved in oxidative stress, cardiac muscle contraction, and cardiac conduction following melphalan treatment and NAC supplementation. As shown in the heatmap (Fig. 7d), the up- and downregulation of genes involved in oxidative stress (e.g., *DUOX2* and *NOX4*) following melphalan treatment (Mel vs. control) was attenuated with NAC supplementation (Mel+NAC vs. control). Similarly, the up- and downregulation of genes involved in cardiac muscle contraction (e.g., Ca^2+^ handling proteins *CACNA1C*, *RYR2*, and *CASQ2* and cardiac contractile proteins *TNNC1*, *ACTC1*, and *TNNC1*) and cardiac conduction (e.g., *ATP2B2* and *ABCC9*) following melphalan treatment (Mel vs. control) was attenuated by NAC supplementation (Mel+NAC vs. control) (Fig. 7d, Fig. S4c).

Finally, we compared the results of proteomics and RNA-Seq analysis. There were 40 genes recognized as DEGs in both analyses, of which 10 were upregulated and 30 were downregulated (Fig. 3b). Intriguingly, 6 of the upregulated genes were involved in the p53 signaling pathway (e.g., *CDKN1A* and *RRM2B*), and 11 of the downregulated genes were relevant to muscle structure (e.g., *TTN* and *TBX20*).

## Discussion

In this study, we found that melphalan caused severe deleterious effects on hiPSC-CMs as indicated by significant cell death, early stage apoptosis, excessive reactive oxygen species, deranged Ca^2+^ handling, and dysfunctional contractility in a dose-dependent fashion. These deleterious effects were attenuated by the treatment of the cells with NAC, a powerful antioxidant, indicating that oxidative stress plays a central role in the mechanism underlying melphalan-induced cardiotoxicity. With the use of hiPSC-CMs as a novel human cell-based model for the characterization of cardiac defects induced by melphalan treatment, we also provide a unique resource of human global transcriptomic and proteomic datasets for melphalan-induced cardiotoxicity, which could be valuable for further investigation of the molecular mechanisms underlying melphalan-induced cardiotoxicity. In particular, our proteomic and transcriptomic analyses also implicated several other signaling pathways including the p53 and TGF-β signaling pathways in melphalan-induced cardiotoxicity.

Oxidative stress in cells results from an imbalance between free radicals that can damage DNA, protein, and cell membrane and antioxidants that can interact with free radicals and prevent their damaging effects [29]. We observed a dose-dependent increase of both intracellular and mitochondria ROS levels following the melphalan treatment of hiPSC-CMs. Consistent with this observation, we also detected increased expression of genes that are known to mediate ROS production such as dual oxidase 2 (*DUOX2*). An increase in the level of ROS was similarly observed in studies of other chemotherapeutic drugs such as doxorubicin [28]. In addition, unlike doxorubicin, melphalan treatment did not suppress the expression of several genes that are important in the endogenous antioxidant defense system including *N*-ribosyldihydronicotinamide: quinone reductase 2 (*NQO2*), superoxide dismutase family of proteins encoding genes (*SODs*), and glutathione producing genes (*GSS*, *GSR*, and *GPX1*). This is not unexpected as proteins that function together in a pathway are likely to evolve in a correlated manner.

Increased oxidative stress in CMs is known to contribute to dysregulation of Ca^2+^ cycling, contractile dysfunction, and arrhythmias [30]. Indeed, the melphalan-induced cardiotoxicity we observed in hiPSC-CMs is associated with not only increased oxidative stress but also abnormal Ca^2+^ handling and reduced contractility. Consistent with these results, we also observed changes in the expression of genes associated with these cellular functions such as genes encoding Ca^2+^ handling proteins, ion transport channels, and contractile proteins. For example, several genes encoding Ca^2+^ handling proteins (e.g., *CACNA1C*, *RYR2*, and *CASQ2*) and cardiac contractile proteins (e.g., *TNNC1*, *ACTC1*, and *TNNC1*) were downregulated following melphalan treatment. These proteins play critical roles in the regulation of cardiac contraction, and their dysregulation can lead to arrhythmias. For example, the dysregulation of *CASQ2*, which is known to amplify the likelihood of diastolic SR Ca^2+^ releases by relieving its inhibitory effects on cardiac-specific ryanodine receptor 2 (RyR2) during diastole, and downregulation of *RYR2* could work collectively to increase the probability of ventricular arrhythmias [31]. Furthermore, while both oxidative stress and abnormal Ca^2+^ handling were observed in melphalan-treated cells, our results also strongly suggest that the melphalan-induced changes in cardiac contractility and gene expression are likely to be the direct consequence of oxidative stress because the melphalan-induced defects were attenuated by NAC supplementation. Our findings are consistent with the role of ROS in regulating cardiac function and mediating changes in genes involved in cardiac muscle contraction. For example, ROS can target genes and proteins of Ca^2+^ handling such as *CACNA1C* on sarcolemma, Ca^2+^ transporting ATPase on SR, and Na^+^/Ca^2+^ exchanger to suppress the Ca^2+^ current [32, 33]. Consequently, SR Ca^2+^ content decreases and diastolic Ca^2+^ leak increases; these changes, along with the decreased expression of genes encoding contractile proteins including *TTN*, *MYH7*, and *MYL2*, synergistically act to reduce contractile force [34, 35]. These findings underscore the importance for further analysis of the action potentials of CMs treated with melphalan, although Ca^2+^ transients are reported to closely reflect action potential characteristics of hiPSC-CMs [36].

Both transcriptomic and proteomic analyses consistently show that melphalan treatment of hiPSC-CMs significantly altered the tumor suppressor p53 signaling pathway, which is an important regulator of the cellular response to genotoxic drugs and oxidative stress-induced DNA damage [37]. The activation of p53 stimulates DNA repair processes; however, if double-strand breaks are not properly repaired, persistent accumulation of p53 can lead to induction of apoptosis in the damaged cells [38]. Apoptosis is well accepted as an important mechanism of anthracycline-induced cardiotoxicity as well [39]. Furthermore, with regard to the p53 signaling pathway, we also found that melphalan treatment remarkably upregulated the expression of *CDKN1A.* This gene encodes p21 which is known to be tightly controlled by p53 to mediate the p53-dependent cell cycle arrest and interact with endogenous antioxidant defense systems in response to a variety of stress stimuli to protect CMs [40]. Together, these observations suggest that the p53 signaling pathway is likely to play a critical role in melphalan-induced cardiotoxicity in hiPSC-CMs.

Our results also show that melphalan treatment of hiPSC-CMs altered the expression of several other signaling pathways related to cell death and diseases. The dramatic downregulation of *THSD4*, *LEFTY2*, and *LTBP1* induced by melphalan treatment could collectively enhance the activation of TGF-β signaling pathway [41] and impact the downstream cellular processes such as the induction of apoptosis as observed in myocardial infarction [42]. It is possible that activation of TGF-β signaling pathway was contributed by ROS and p53, similar to the observation described in ibrutinib- and doxorubicin-induced cardiotoxicity [43, 44]. Furthermore, melphalan treatment of hiPSC-CMs also altered the expression of genes associated with cytokine-cytokine receptor interaction which can regulate and mediate various signaling pathways including TGF-β signaling. For example, we found that melphalan treatment resulted in a 20-fold increase in the expression of *GDF15*, which is a secreted ligand of the TGF-β superfamily of proteins that can activate the canonical TGF-β signaling to regulate cell cycle [45] and can be also induced by p53 to act as a growth inhibitory molecule [46]. Consistent with the role of TGF-β signaling in the cellular stress response in disease conditions such as inflammation and acute injury [47], melphalan also affected the expression of several genes encoding the tumor necrosis factor (TNF) superfamily and the TNF receptor superfamily (TNFRSF) proteins, which are associated with inflammation and tissue injury. Specifically, melphalan upregulated the expression of genes encoding all subunits of TNFRSF10, which are known to transduce cell death signal and induce cell apoptosis [48].

hiPSC-CMs have been shown to be an excellent tool to study drug-induced cardiotoxicity, and the use of hiPSC-CMs to detect drug-induced proarrhythmic effects has been demonstrated as part of the evolving Comprehensive in Vitro Proarrhythmia Assay (CiPA) paradigm [49]. We note that compared with adult CMs, hiPSC-CMs lack a fully mature phenotype with smaller and round shape, being mononucleated, and with disorganized sarcomeres. However, despite these differences, hiPSC-CMs express the central components for excitation-contraction coupling, membrane voltage regulation, and Ca^2+^ release and uptake, which are crucial for CM functional studies [50]. Consequently, we believe that our findings are likely to be relevant to the clinically observed cardiotoxicity in patients receiving melphalan treatment. However, for example, although we found that both of p53 and TGF-β signaling pathways likely contributed to melphalan-induced cardiotoxicity, whether targeting each single pathway specifically can adequately protect CMs requires further investigation. Nevertheless, our findings provide molecular insights for further exploiting underlying mechanisms and discovering novel therapeutics.

Finally, NAC effectively reduced oxidative stress and cell death in melphalan-treated hiPSC-CMs. This finding is consistent with accumulating evidence in cell and animal models regarding the role of antioxidants in preventing antineoplastic drug-induced cardiotoxicity and oxidative stress-induced cardiomyopathy. For instance, therapeutic inhibition of ROS by mito-TEMPO and vitamin C was found to reduce adverse cardiac changes in diabetic cardiomyopathy and anthracycline-induced cardiotoxicity [51, 52]. NAC, as an important source of reduced glutathione and sulfhydryl groups, can directly interact with free radicals in cells [53]. It is an FDA-approved medical supplement and has been applied in oxidative stress-induced diseases such as acetaminophen-induced hepatotoxicity, chronic bronchitis, ulcerative colitis, asthma, Alzheimer, and Parkinson [54]. Due to its proven safety and efficacy, NAC may have promising therapeutic value in treating melphalan-induced cardiotoxicity.

## Conclusions

In summary, our study has demonstrated that the clinically observed cardiotoxicity of melphalan can be recapitulated in the model of hiPSC-CMs. Melphalan treatment of hiPSC-CMs induces oxidative stress, apoptosis and cell death, deranged Ca^2+^ handling, dysfunctional contractility, and alterations of global transcriptomic and proteomic profiles. In addition, we have found that NAC can attenuate these deleterious effects of melphalan treatment in hiPSC-CMs, indicating that oxidative stress plays a central role in melphalan-induced cardiotoxicity.

## Supplementary Information

**Additional file 1: Fig. S1.** Directed differentiation of hiPSCs and generation of highly enriched hiPSC-CMs. **Fig. S2.** Validation of CellTiter-Blue and CellTiter-Glo 3D Cell Viability Assays. **Fig. S3.** Melphalan treatment of hiPSC-CMs induces oxidative stress. **Fig. S4.** NAC attenuates melphalan-induced alteration of hiPSC-CM transcriptome profiles characterized by RNA-Seq analysis. **Fig. S5.** Melphalan treatment does not alter hiPSC-CM purity. **Table S1.** Information of major reagents. **Table S2.** Antibodies for immunocytochemistry. **Table S3.** SyBr green primers for qRT-PCR. **Table S4**. List of top 20 DEGs and enriched GO terms in hiPSC-CMs treated with melphalan compared with no melphalan treatment based on proteomic analysis. **Table S5**. List of top 20 DEGs, enriched GO terms and KEGG pathways in hiPSC-CMs treated with melphalan compared with no melphalan treatment based on RNA-Seq analysis. **Table S6**. List of top 20 DEGs, enriched GO terms and KEGG pathways in melphalan-treated hiPSC-CMs with NAC supplementation compared with no supplementation based on RNA-Seq analysis.

## Data Availability

The proteomics data is available at PeptideAtlas repository (PASS01576) and RNA-Seq data is available at GEO repository (GSE150055).

## Abbreviations

hiPSC-CMs: Human induced pluripotent stem cells
CMs: Cardiomyocytes
RNA-Seq: RNA-Sequencing
RT: Room temperature
NAC: *N*-Acetyl-l-cysteine
qRT-PCR: Quantitative real-time polymerase chain reaction
GO: Gene Ontology
DEGs: Differentially expressed genes
ROS: Reactive oxygen species
FPKM: Fragments Per Kilobase Million
MFI: Mean fluorescence intensity
TGF: Transforming growth factor
DUOX2: Dual oxidase 2
NQO2: *N*-Ribosyldihydronicotinamide: quinone reductase 2
SOD: Superoxide dismutase
RyR: Ryanodine receptor
TNF: Tumor necrosis factor
TNFRSF: TNF receptor superfamily
