# Supplementary material for: Melphalan induces cardiotoxicity through oxidative stress in cardiomyocytes derived from human induced pluripotent stem cells
Source: Stem Cell Res Ther. 2020 Nov 5;11:470. doi: 10.1186/s13287-020-01984-1 (PMC7643439; doi:10.1186/s13287-020-01984-1)
Supplement: Supplementary file 1 — Additional file 1: Fig. S1. Directed differentiation of hiPSCs and generation of highly enriched hiPSC-CMs. Fig. S2. Validation of CellTiter-Blue and CellTiter-Glo 3D Cell Viability Assays. Fig. S3. Melphalan treatment of hiPSC-CMs induces oxidative stress. Fig. S4. NAC attenuates melphalan-induced alteration of hiPSC-CM transcriptome profiles characterized by RNA-Seq analysis. Fig. S5. Melphalan treatment does not alter hiPSC-CM purity. Table S1. Information of major reagents. Table S2. Antibodies for immunocytochemistry. Table S3. SyBr green primers for qRT-PCR. Table S4. List of top 20 DEGs and enriched GO terms in hiPSC-CMs treated with melphalan compared with no melphalan treatment based on proteomic analysis. Table S5. List of top 20 DEGs, enriched GO terms and KEGG pathways in hiPSC-CMs treated with melphalan compared with no melphalan treatment based on RNA-Seq analysis. Table S6. List of top 20 DEGs, enriched GO terms and KEGG pathways in melphalan-treated hiPSC-CMs with NAC supplementation compared with no supplementation based on RNA-Seq analysis. [file 13287_2020_1984_MOESM1_ESM.pdf]

*Supplementary Information*

# Melphalan induces cardiotoxicity through oxidative stress in cardiomyocytes derived from human induced pluripotent stem cells

Rui Liu, Dong Li, Fangxu Sun, Antonio Rampoldi, Joshua T. Maxwell, Ronghu Wu, Peter Fischbach, Sharon M. Castellino, Yuhong Du, Haian Fu, Anant Mandawat, and Chunhui Xu

Correspondence:

Chunhui Xu, PhD, Associate Professor, Department of Pediatrics, Emory University School of Medicine, 2015 Uppergate Drive, Atlanta, GA 30322, USA. Email: chunhui.xu@emory.edu

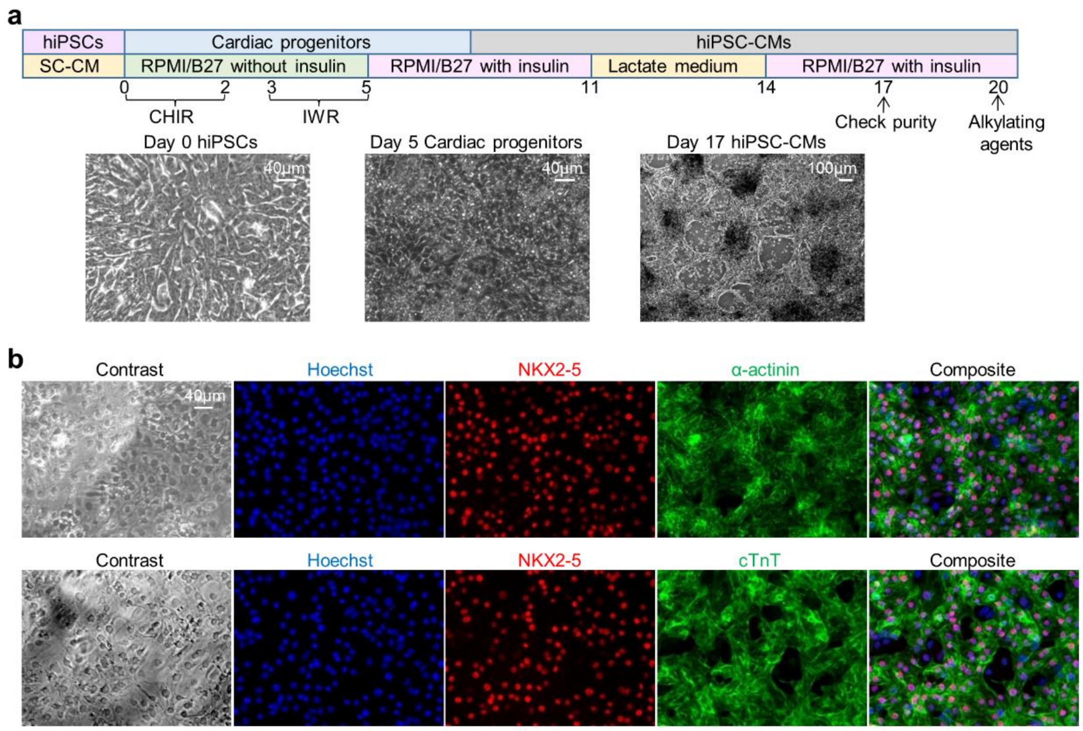


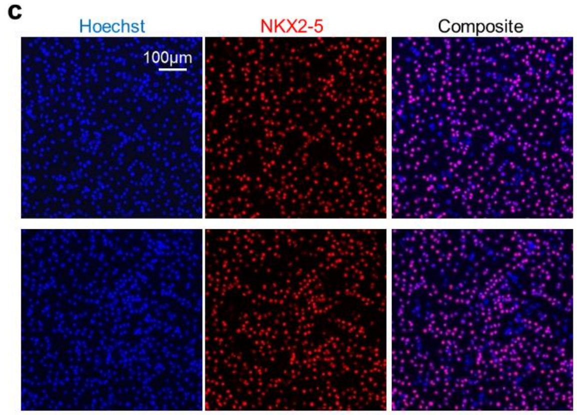


| Replicate Well NO. | Nucleus Count | NKX2-5 Positive Object Count | % hiPSC-CMs |
|---|---|---|---|
| 1 | 10937 | 9860 | 90.2% |
| 2 | 10813 | 9768 | 90.3% |
| 3 | 10951 | 9839 | 89.8% |
| 4 | 10530 | 9387 | 89.1% |
| 5 | 10786 | 9655 | 89.5% |
| Average | 10803 | 9702 | 89.8% |

**Fig. S1 Directed differentiation of hiPSCs and generation of highly enriched hiPSC-CMs. a** hiPSCs were induced for CM differentiation and hiPSC-CMs were enriched by metabolic selection. A parallel culture of hiPSC-CMs was harvested to determine CM purity at day 17, and the rest of the cells were cultured until day 20 for subsequent assessments. **b** Representative images of immunocytochemistry revealing the majority of the cells in culture were positive for cardiac transcription factor NKX2-5, and structural proteins cardiac troponin T and α-actinin at day 17. **c** Representative images acquired from ArrayScan and quantitative summary of percentage of NKX2-5-positive cells (~90%) indicated highly enriched CMs were generated in the cultures at day 17. SC-CM, stem cell culture medium.

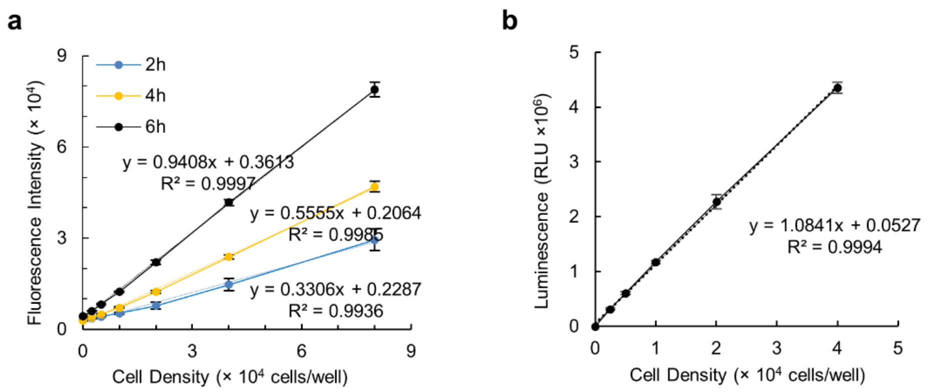


**Fig. S2 Validation of CellTiter-Blue and CellTiter-Glo 3D Cell Viability Assays.** hiPSC-CMs were seeded at a range of numbers from 0.25 to 4 or 8 × $10^4$ cells per well, cultured for 24 h, and then incubated the cells with CellTiter-Blue reagent for 2, 4, and 6 h or CellTiter-Glo 3D reagent, respectively. **a** Representative plot presenting the linear relationship between hiPSC-CM numbers and fluorescence intensity via CellTiter-Blue Viability Assay (n = 5). **b** Representative plot presenting the linear relationship between hiPSC-CM numbers and luminescence intensity of via CellTiter-Glo 3D Viability Assay (n = 5). Note: The readout of fluorescence or luminescence intensity had a nearly linear relationship with the number of cells ($R^2 > 0.99$), suggesting that these two assays were reliable and sensitive for the estimation of cell numbers of hiPSC-CMs.

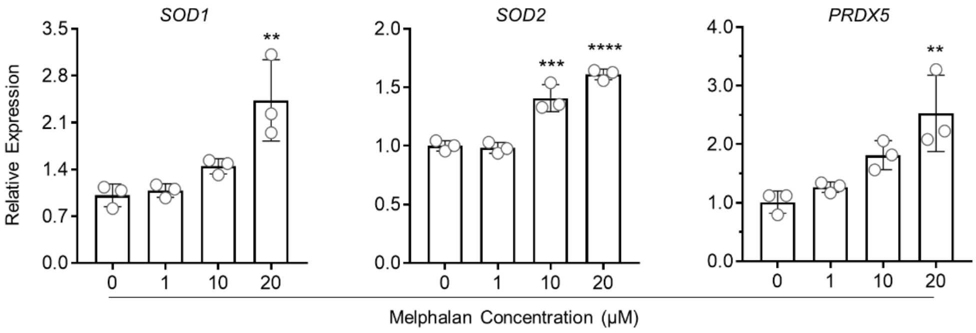


**Fig. S3 Melphalan treatment of hiPSC-CMs induces oxidative stress.** qRT-PCR analysis showing relative gene expression levels of oxidative stress-related genes including *SOD1*, *SOD2*, and *PRDX5* in hiPSC-CMs treated with melphalan for 3 days (n = 3). Comparisons were conducted between each treatment group and no melphalan group via One-way ANOVA test. **, *P*-value < 0.01; ***, *P*-value < 0.001; ****, *P*-value < 0.0001.

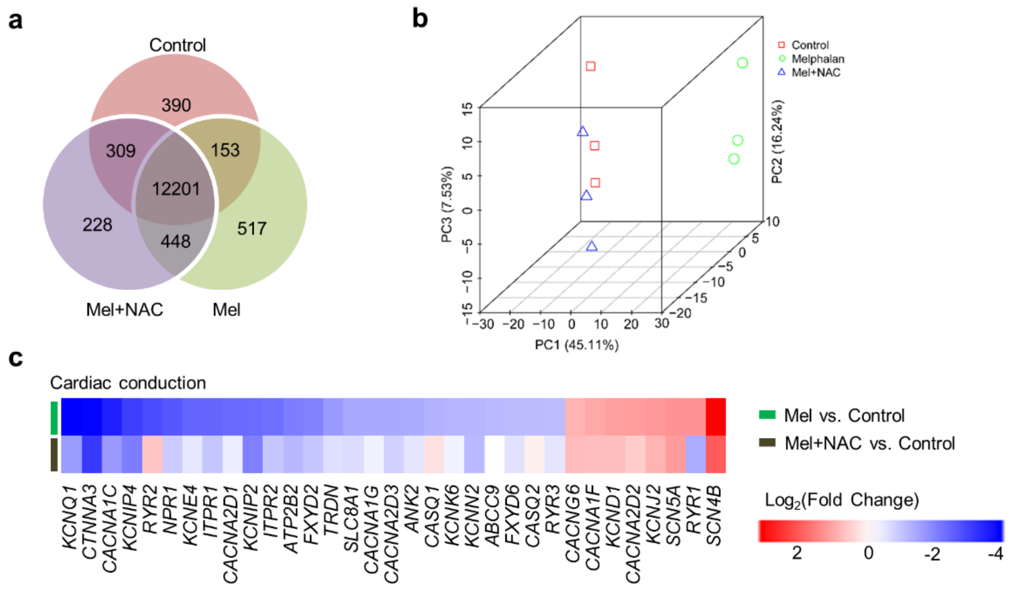


**Fig. S4 NAC attenuates melphalan-induced alteration of hiPSC-CM transcriptome profiles characterized by RNA-Seq analysis.** RNA-Seq analysis of hiPSC-CMs upon 0 and 20 μM of melphalan treatment with or without NAC supplementation for 3 days (n = 3). **a** Venn diagram showing the amounts of commonly and uniquely expressed genes among different treatment groups. **b** Principal component analysis plot showing the differences between groups and the distribution of samples in each group. **c** Heatmap showing the DEGs involved in GO term of cardiac conduction in melphalan- or Mel+NAC-treated hiPSC-CMs compared with control group, respectively. Blue and red colors of displayed rectangles indicate the levels of gene expression according to $\log_2$(fold change). Control, no melphalan; Mel, 20 μM melphalan; Mel+NAC, 20 μM melphalan with 1 mM NAC.

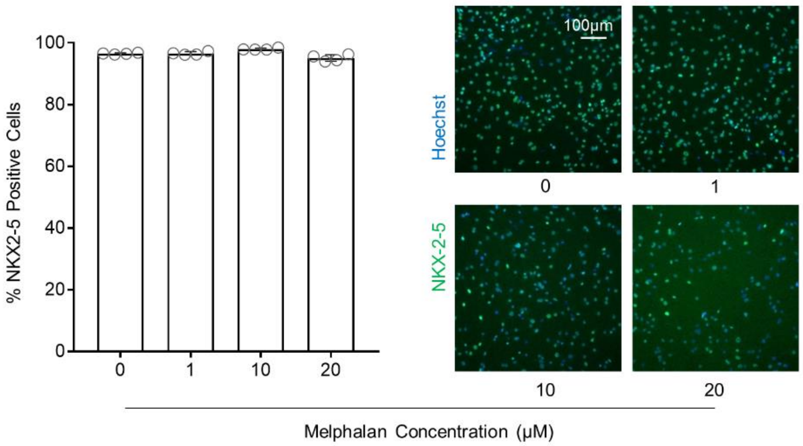


**Fig. S5 Melphalan treatment does not alter hiPSC-CM purity.** hiPSC-CM purity was determined via ArrayScan. Cells were fixed and stained with first and secondary antibodies to detect NKX2-5. Nuclei were stained with Hoechst upon melphalan treatment for 5 days. NKX2-5-positive cells emitted bright green nuclear fluorescence. Representative images and quantification of percentage of NKX2-5-positive cells were shown (n = 4). Comparisons were conducted between each treatment group and no melphalan group via One-way ANOVA test.

**Table S1.** Information of major reagents

| Product Name | Supplier | Catalog# |
|---|---|---|
| mTeSR1 defined medium | Stem Cell Technologies | 85850 |
| Versene | Thermo Fisher Scientific | 15040066 |
| RPMI 1640 medium | Thermo Fisher Scientific | 11875093 |
| B27 Supplement (50×), minus insulin | Thermo Fisher Scientific | A1895601 |
| B27 Supplement (50×), serum free | Thermo Fisher Scientific | 17504044 |
| RPMI1640, no glucose | Thermo Fisher Scientific | 11879020 |
| Sodium DL-lactate solution | Sigma | L4263 |
| Matrigel | Thermo Fisher Scientific | CB40230C |
| CHIR99021 | Selleckchem | S2924 |
| IWR1 | Sigma | I0161 |
| paraformaldehyde | Electron Microscopy Sciences | 15710 |
| Hoechst33342 | Thermo Fisher Scientific | H3570 |
| melphalan | Selleckchem | S8266 |
| dimethyl sulfoxide | Sigma | D2438 |
| N-Acetyl-L-cysteine | Sigma | A9165 |
| fetal bovine serum | GE Healthcare Life Sciences, Hyclone Laboratories | SH30396.03 |
| Live Cell Imaging Solution | Thermo Fisher Scientific | A14291DJ |
| CellTiter-Blue Cell Viability Assay | Promega | G8081 |
| CellTiter-Glo 3D Cell Viability Assay | Promega | G9683 |
| CellEvent Caspase-3/7 Green Detection Reagent | Thermo Fisher Scientific | C10423 |
| Image-iT LIVE Green Reactive Oxygen Species Detection Kit (DCFDA) | Thermo Fisher Scientific | I36007 |
| MitoSOX Red Mitochondrial Superoxide Indicator | Thermo Fisher Scientific | M36008 |
| Fluo-4, AM | Thermo Fisher Scientific | F14201 |
| Aurum total RNA mini kit | Bio-Rad | 732-6820 |
| SuperScript VILO cDNA Synthesis Kit | Thermo Fisher Scientific | 11754050 |
| iTaq SyBr green master mix | Bio-Rad | 172-5121 |

**Table S2.** Antibodies for immunocytochemistry

| Type | Target | Isotype | Supplier | Catalog# | Dilution |
|---|---|---|---|---|---|
| Primary | α-actinin | mouse $IgG_1$ | Sigma | A7811 | 1:800 |
| | NKX2-5 | rabbit IgG | Cell Signaling Technologies | SC14033 | 1:1600 |
| | cardiac troponin T | mouse $IgG_1$ | Fisher Scientific | MS295P1 | 1:200 |
| Secondary | Alexa 488, Goat anti-mouse IgG1 | | Invitrogen | A-21121 | 1:1000 |
| | Alexa 594, Goat anti-rabbit IgG | | Invitrogen | A-11012 | 1:1000 |
| | Alexa 488, Goat anti-rabbit IgG | | Invitrogen | A-11034 | 1:1000 |

**Table S3.** SyBr green primers for qRT-PCR

| Gene | Full name | Accession code | Primer |
|---|---|---|---|
| *BCL2* | B-cell CLL/lymphoma 2 | NM_000633.2 | Forward: GAGAAATCAAACAGAGGCCG Reverse: CTGAGTACCTGAACCGGCA |
| *BAX* | BCL2-associated X protein | NM_004324 | Forward: GGAGGAAGTCCAATGTCCAG Reverse: TCTGACGGCAACTTCAACTG |
| *SOD1* | Superoxide dismutase 1 | NM_000454 | Forward: GGTGGGCCAAAGGATGAAGAG Reverse: CCACAAGCCAAACGACTTCC |
| *SOD2* | Superoxide dismutase 2 | NM_000636 | Forward: GCTCCGGTTTTGGGGTATCTG Reverse: GCGTTGATGTGAGGTTCCAG |
| *SOD3* | Superoxide dismutase 3 | NM_003102 | Forward: ATGCTGGCGCTACTGTGTTC Reverse: CTCCGCCGAGTCAGAGTTG |
| *GSR* | Glutathione reductase | NM_001195102 | Forward: CACTTGCGTGAATGTTGGATG Reverse: TGGGATCACTCGTGAAGGCT |
| *NQO2* | NAD(P)H dehydrogenase, quinone 2 | NM_000904 | Forward: GTACTCATTGTCTATGCACACCA Reverse: TGCCTGCTCAGTTCATCTACA |
| *GPX1* | Glutathione peroxidase 1 | NM_201397 | Forward: CAGTCGGTGTATGCCTTCTCG Reverse: GAGGGACGCCACATTCTCG |
| *PRDX5* | Peroxiredoxin 5 | NM_181651 | Forward: TCCTGGCTGATCCCACTGG |

| | | | |
|---|---|---|---|
| | | | Reverse: CTGTGAGATGATATTGGGTGCC |
| *RYR2* | Ryanodine receptor 2 | NM_001035 | Forward: CAAATCCTTCTGCTGCCAAG Reverse: CGAAGACGAGATCCAGTTCC |
| *CACNA1C* | Calcium channel, voltage-dependent, L type, alpha 1C subunit | NM_000719 | Forward: TTTTAAAAACGCTTCCACCG Reverse: TTCCAGAAGATGATTCCAACG |
| *TNNI1* | Troponin I type 1 | NM_003281 | Forward: AGCATCAGGCTCTTCAGCA Reverse: ACAGTCTGCAGTCTACGGCG |
| *TNNT2* | Troponin T type 2 | NM_001001431 | Forward: GCGGGTCTTGGAGACTTTCT Reverse: TTCGACCTGCAGGAGAAGTT |
| *MYH6* | Myosin heavy chain 6 | NM_002471 | Forward: CTTCTCCACCTTAGCCCTGG Reverse: GCTGGCCCTTCAACTACAGA |
| *MYH7* | Myosin heavy chain 7 | NM_000257 | Forward: CGCACCTTCTTCTCTTGCTC Reverse: GAGGACAAGGTCAACACCCT |
| *MYL2* | Myosin light chain 2 | NM_000432 | Forward: CGTTCTTGTCAATGAAGCCA Reverse: CAACGTGTTCTCCATGTTCG |
| *MYL7* | Myosin light chain 7 | NM_021223 | Forward: CTTGTAGTCGATGTTCCCCG Reverse: TCAAGCAGCTTCTCCTGACC |

| | | | |
|---|---|---|---|
| *GAPDH* | Glyceraldehyde-3-phosphate dehydrogenase | NM_001256799 | Forward: CTGGGCTACACTGAGCACC<br>Reverse: AAGTGGTCGTTGAGGGCAATG |

**Table S4.** List of top 20 DEGs and enriched GO terms in hiPSC-CMs treated with melphalan compared with no melphalan treatment based on proteomic analysis.

| Gene Symbol | Gene Description | Fold Change | -Log$_{10}$($P$-value) |
|---|---|---|---|
| Up-regulated | | | |
| *TNFRSF10C* | Tumor necrosis factor receptor superfamily member 10c | 3.900 | 3.14286 |
| *HBD* | Hemoglobin Subunit Delta | 2.974 | 1.88666 |
| *DDB2* | Damage Specific DNA Binding Protein 2 | 2.963 | 2.81423 |
| *HBA1* | Hemoglobin Subunit Alpha 1 | 2.445 | 1.81212 |
| *RRM2* | Ribonucleotide Reductase Regulatory Subunit M2 | 2.391 | 2.16014 |
| *CDKN1A* | Cyclin-dependent kinase inhibitor 1A (p21 Cip1) | 2.383 | 1.65025 |
| *NFE2L2* | Nuclear Factor, Erythroid 2 Like 2 | 2.271 | 1.82614 |
| *AMOT* | Angiomotin | 2.194 | 2.00372 |
| *APOA1* | Apolipoprotein A1 | 2.193 | 1.84905 |
| *S100A13* | S100 Calcium Binding Protein A13 | 2.178 | 2.20418 |
| Down-regulated | | | |
| *MRPS18A* | Mitochondrial Ribosomal Protein S18A | 0.601 | 4.26649 |
| *RPL32* | Ribosomal Protein L32 | 0.424 | 4.03046 |
| *TRIM24* | Tripartite Motif Containing 24 | 0.661 | 4.00693 |
| *HNRNPD* | Heterogeneous Nuclear Ribonucleoprotein D | 0.385 | 3.79572 |
| *PNN* | Pinin, Desmosome Associated Protein | 0.653 | 3.56729 |
| *TBX20* | T-Box Transcription Factor 20 | 0.419 | 3.39066 |
| *PRKCA* | Protein Kinase C Alpha | 0.572 | 3.24993 |
| *STXBP6* | Syntaxin Binding Protein 6 | 0.638 | 3.09285 |
| *RPL19* | Ribosomal Protein L19 | 0.422 | 3.07857 |
| *RPL8* | Ribosomal Protein L8 | 0.457 | 3.06067 |
| GO Term ID | GO Term Description | Gene Count | $P$-value |
| Up-regulated | | | |
| GO:0051346 | Negative regulation of hydrolase activity | 16 | 1.20842E-11 |
| GO:0043086 | Negative regulation of catalytic activity | 20 | 8.6046E-11 |
| GO:0050819 | Negative regulation of coagulation | 8 | 5.81985E-10 |
| GO:0042060 | Wound healing | 16 | 8.1444E-10 |

| | | | |
|---|---|---|---|
| GO:0061041 | Regulation of wound healing | 10 | 9.43747E-10 |
| GO:0009611 | Response to wounding | 17 | 1.07669E-09 |
| GO:0044092 | Negative regulation of molecular function | 21 | 1.16815E-09 |
| GO:0050818 | Regulation of coagulation | 9 | 1.20956E-09 |
| GO:1903034 | Regulation of response to wounding | 10 | 3.22653E-09 |
| GO:0007596 | Blood coagulation | 13 | 3.64145E-09 |
| Down-regulated | | | |
| GO:0003735 | Structural constituent of ribosome | 50 | 3.63833E-53 |
| GO:0005198 | Structural molecule activity | 58 | 2.19188E-34 |
| GO:0044822 | Poly(A) RNA binding | 67 | 6.16268E-33 |
| GO:0003723 | RNA binding | 73 | 1.16359E-29 |
| GO:0003676 | Nucleic acid binding | 97 | 2.70215E-20 |
| GO:1901363 | Heterocyclic compound binding | 107 | 1.09761E-13 |
| GO:0097159 | Organic cyclic compound binding | 107 | 2.96819E-13 |
| GO:0019843 | rRNA binding | 11 | 4.7287E-10 |
| GO:0050839 | Cell adhesion molecule binding | 19 | 7.47353E-07 |
| GO:0098641 | Cadherin binding involved in cell-cell adhesion | 15 | 1.16713E-06 |

**Table S5.** List of top 20 DEGs, enriched GO terms and KEGG pathways in hiPSC-CMs treated with melphalan compared with no melphalan treatment based on RNA-Seq analysis.

| Gene Symbol | Gene Description | $Log_2$(Fold Change) | Adjusted *P*-value |
|---|---|---|---|
| Up-regulated | | | |
| *GDF15* | Growth differentiation factor 15 | 4.4469 | 3.69E-268 |
| *CDKN1A* | Cyclin-dependent kinase inhibitor 1A (p21 Cip1) | 4.224 | 0 |
| *DUSP13* | Dual specificity phosphatase 13 | 3.9975 | 1.01E-33 |
| *GRHL3* | Grainyhead-like transcription factor 3 | 4.3444 | 7.80E-27 |
| *DRAXIN* | Dorsal inhibitory axon guidance protein | 3.948 | 6.61E-33 |
| *ZSCAN4* | Zinc finger and SCAN domain containing 4 | 3.8087 | 1.33E-19 |
| *FDXR* | Ferredoxin reductase | 3.798 | 0 |
| *TNFRSF10C* | Tumor necrosis factor receptor superfamily member 10c | 3.729 | 1.65E-138 |
| *SCN4B* | Sodium channel voltage gated type IV beta subunit | 3.6045 | 2.96E-29 |
| *BPIFA1* | BPI fold containing family A member 1 | 3.5181 | 3.98E-14 |
| Down-regulated | | | |
| *GPC6* | Glypican 6 | -6.3842 | 0 |
| *CNTN1* | Contactin 1 | -6.0072 | 3.36E-74 |
| *SDK1* | Sidekick cell adhesion molecule 1 | -5.7863 | 4.10E-81 |
| *CDH13* | Cadherin 13 | -5.5657 | 1.47E-101 |
| *THSD4* | Thrombospondin type 1 domain containing 4 | -5.5175 | 1.50E-199 |
| *PARK2* | Parkin RBR E3 ubiquitin protein ligase | -5.4659 | 4.31E-48 |
| *SLC24A3* | Solute carrier family 24 member 3 | -5.4454 | 6.87E-50 |
| *MSRA* | Methionine sulfoxide reductase A | -5.2313 | 1.21E-63 |
| *PARD3B* | Par-3 family cell polarity regulator beta | -5.2087 | 5.45E-75 |
| *CTNND2* | Catenin delta 2 | -5.0364 | 1.20E-108 |
| GO Term ID | GO Term Description | Gene Count | Adjusted *P*-value |
| Down-regulated | | | |
| GO:0030198 | extracellular matrix organization | 93 | 2.62E-23 |
| GO:0043062 | extracellular structure organization | 96 | 8.13E-21 |
| GO:0006936 | muscle contraction | 78 | 3.10E-15 |

| | | | |
|---|---|---|---|
| GO:0003012 | muscle system process | 83 | 5.42E-12 |
| GO:0007416 | synapse assembly | 47 | 7.21E-12 |
| GO:0060047 | heart contraction | 58 | 3.44E-10 |
| GO:0003015 | heart process | 59 | 3.44E-10 |
| GO:0050808 | synapse organization | 74 | 3.44E-10 |
| GO:0030199 | collagen fibril organization | 22 | 9.01E-10 |
| GO:0051965 | positive regulation of synapse assembly | 25 | 1.69E-09 |
| GO:0061448 | connective tissue development | 55 | 2.67E-09 |
| GO:0060537 | muscle tissue development | 70 | 4.90E-09 |
| GO:1903522 | regulation of blood circulation | 57 | 4.90E-09 |
| GO:1904018 | positive regulation of vasculature development | 45 | 4.90E-09 |
| GO:0051963 | regulation of synapse assembly | 31 | 7.11E-09 |
| GO:0051216 | cartilage development | 45 | 1.28E-08 |
| GO:0034765 | regulation of ion transmembrane transport | 77 | 1.56E-08 |
| GO:0050804 | modulation of chemical synaptic transmission | 73 | 1.59E-08 |
| GO:0099177 | regulation of trans-synaptic signaling | 73 | 1.68E-08 |
| GO:0031589 | cell-substrate adhesion | 63 | 2.18E-08 |
| KEGG Pathway ID | KEGG Pathway Description | Gene Count | Adjusted *P*-value |
| hsa04512 | ECM-receptor interaction | 28 | 5.26E-06 |
| hsa04020 | Calcium signaling pathway | 47 | 5.26E-06 |
| hsa05414 | Dilated cardiomyopathy (DCM) | 29 | 5.82E-06 |
| hsa04115 | p53 signaling pathway | 25 | 7.86E-06 |
| hsa05410 | Hypertrophic cardiomyopathy (HCM) | 27 | 7.86E-06 |
| hsa04261 | Adrenergic signaling in cardiomyocytes | 36 | 7.28E-05 |
| hsa04974 | Protein digestion and absorption | 25 | 0.000136 |
| hsa04510 | Focal adhesion | 44 | 0.000145 |
| hsa04270 | Vascular smooth muscle contraction | 32 | 0.00023 |
| hsa05412 | Arrhythmogenic right ventricular cardiomyopathy (ARVC) | 22 | 0.00023 |
| hsa04911 | Insulin secretion | 24 | 0.000236 |
| hsa04010 | MAPK signaling pathway | 56 | 0.000269 |
| hsa04080 | Neuroactive ligand-receptor interaction | 57 | 0.000269 |

| | | | |
|---|---|---|---|
| hsa04151 | PI3K-Akt signaling pathway | 61 | 0.000269 |
| hsa04933 | AGE-RAGE signaling pathway in diabetic complications | 26 | 0.000269 |
| hsa04514 | Cell adhesion molecules (CAMs) | 30 | 0.000269 |
| hsa04360 | Axon guidance | 40 | 0.000277 |
| hsa04925 | Aldosterone synthesis and secretion | 25 | 0.000381 |
| hsa04670 | Leukocyte transendothelial migration | 27 | 0.000495 |
| hsa04015 | Rap1 signaling pathway | 42 | 0.00054 |

**Table S6.** List of top 20 DEGs, enriched GO terms and KEGG pathways in melphalan-treated hiPSC-CMs with NAC supplementation compared with no supplementation based on RNA-Seq analysis.

| Gene Symbol | Gene Description | $Log_2$(Fold Change) | Adjusted *P*-value |
|---|---|---|---|
| Up-regulated | | | |
| *GPC6* | Glypican 6 | 3.5211 | 7.20E-97 |
| *THSD4* | Thrombospondin type 1 domain containing 4 | 3.3196 | 1.51E-65 |
| *SMOC2* | SPARC related modular calcium binding 2 | 3.1634 | 3.82E-34 |
| *SPOCK1* | Sparc/osteonectin cwcv and kazal-like domains proteoglycan (testican) 1 | 3.0824 | 1.03E-214 |
| *LEFTY2* | Left-right determination factor 2 | 3.0782 | 1.03E-223 |
| *COL21A1* | Collagen type XXI alpha 1 | 2.9993 | 3.31E-68 |
| *FGF1* | Fibroblast growth factor 1 (acidic) | 2.9685 | 1.46E-48 |
| *SORCS3* | Sortilin-related VPS10 domain containing receptor 3 | 2.9096 | 2.93E-55 |
| *ACTA1* | Actin alpha 1 skeletal muscle | 2.8581 | 1.07E-201 |
| *LMOD2* | Leiomodin 2 (cardiac) | 2.8378 | 4.30E-205 |
| Down-regulated | | | |
| *ZSCAN4* | Zinc finger and SCAN domain containing 4 | -2.1307 | 1.51E-14 |
| *SCN2A* | Sodium channel voltage gated type II alpha subunit | -2.0074 | 2.82E-28 |
| *GRIN2C* | Glutamate receptor ionotropic N-methyl D-aspartate 2C | -1.6951 | 1.09E-10 |
| *CEND1* | Cell cycle exit and neuronal differentiation 1 | -1.5414 | 2.87E-40 |
| *DRAXIN* | Dorsal inhibitory axon guidance protein | -1.5412 | 5.01E-13 |
| *VGF* | VGF nerve growth factor inducible | -1.5388 | 4.13E-08 |
| *TRIM49C* | Tripartite motif containing 49C | -1.5274 | 1.28E-07 |
| *TAF11L11* | TATA-Box Binding Protein Associated Factor 11 Like 11 | -1.5038 | 2.90E-07 |
| *P2RX6* | Purinergic receptor P2X ligand gated ion channel 6 | -1.4743 | 2.58E-13 |
| *KLLN* | Killin, p53-regulated DNA replication inhibitor | -1.4282 | 9.33E-29 |
| GO Term ID | GO Term Description | Gene Count | Adjusted *P*-value |
| Up-regulated | | | |
| GO:0030198 | extracellular matrix organization | 66 | 1.10E-29 |
| GO:0043062 | extracellular structure organization | 68 | 4.73E-28 |
| GO:0030199 | collagen fibril organization | 18 | 2.72E-12 |

| | | | |
|---|---|---|---|
| GO:0006936 | muscle contraction | 43 | 4.60E-12 |
| GO:0007517 | muscle organ development | 44 | 5.82E-11 |
| GO:0031589 | cell-substrate adhesion | 41 | 7.67E-11 |
| GO:0003012 | muscle system process | 46 | 9.16E-11 |
| GO:0010810 | regulation of cell-substrate adhesion | 30 | 6.84E-10 |
| GO:0003015 | heart process | 33 | 1.23E-08 |
| GO:0030239 | myofibril assembly | 17 | 1.29E-08 |
| GO:1903522 | regulation of blood circulation | 33 | 1.79E-08 |
| GO:0060047 | heart contraction | 32 | 1.93E-08 |
| GO:0008016 | regulation of heart contraction | 29 | 9.10E-08 |
| GO:0061448 | connective tissue development | 30 | 2.25E-07 |
| GO:0055002 | striated muscle cell development | 23 | 2.39E-07 |
| GO:0010927 | cellular component assembly involved in morphogenesis | 19 | 2.69E-07 |
| GO:0060537 | muscle tissue development | 36 | 8.84E-07 |
| GO:0055001 | muscle cell development | 23 | 9.21E-07 |
| GO:0070252 | actin-mediated cell contraction | 18 | 2.04E-06 |
| GO:0090130 | tissue migration | 30 | 2.06E-06 |
| KEGG Pathway ID | KEGG Pathway Description | Gene Count | Adjusted *P*-value |
| hsa04933 | AGE-RAGE signaling pathway in diabetic complications | 20 | 2.01E-08 |
| hsa04020 | Calcium signaling pathway | 27 | 2.07E-08 |
| hsa04974 | Protein digestion and absorption | 18 | 2.46E-08 |
| hsa04512 | ECM-receptor interaction | 16 | 9.40E-07 |
| hsa04270 | Vascular smooth muscle contraction | 19 | 3.22E-06 |
| hsa04510 | Focal adhesion | 24 | 3.22E-06 |
| hsa04925 | Aldosterone synthesis and secretion | 15 | 2.95E-05 |
| hsa05410 | Hypertrophic cardiomyopathy (HCM) | 14 | 2.95E-05 |
| hsa04066 | HIF-1 signaling pathway | 14 | 0.000107 |
| hsa04151 | PI3K-Akt signaling pathway | 28 | 0.000141 |
| hsa04921 | Oxytocin signaling pathway | 18 | 0.000141 |
| hsa04022 | cGMP-PKG signaling pathway | 18 | 0.000232 |
| hsa05414 | Dilated cardiomyopathy (DCM) | 13 | 0.000232 |
| hsa04911 | Insulin secretion | 12 | 0.000519 |

| | | | |
|---|---|---|---|
| hsa05146 | Amoebiasis | 12 | 0.000782 |
| hsa04010 | MAPK signaling pathway | 24 | 0.000782 |
| hsa04350 | TGF-beta signaling pathway | 12 | 0.001084 |
| hsa05205 | Proteoglycans in cancer | 18 | 0.001737 |
| hsa04924 | Renin secretion | 10 | 0.001737 |
| hsa05412 | Arrhythmogenic right ventricular cardiomyopathy (ARVC) | 10 | 0.002371 |
